# Analytical and machine learning approaches identify a sea star steroid with promising activity for COVID-19 therapeutic development

**DOI:** 10.1038/s41598-025-20443-6

**Published:** 2025-10-07

**Authors:** Mohamed S. M. Abd El Hafez, Aya I. Maiyza, Hanan A. Hassan, Sohila Osama, Mohamed G. Seadawy, Maha A. El Demellawy, Doaa A. Ghareeb

**Affiliations:** 1 National Institute of Oceanography and Fisheries, NIOF, Cairo, Egypt; 2 Informatics Research Institute (IRI), City of Scientific Research and Technological Applications (SRTA-City), Alexandria, Egypt; 3 Institute of Graduate Studies and Research, Alexandria University, Alexandria, Egypt; 4 Biodefense center for infectious and emerging diseases, MOD, Cairo, Egypt; 5 Medical Biotechnology Department (MBD), Genetic Engineering and Biotechnology Research Institute (GEBRI), City of Scientific Research and Technological Applications (SRTA-City), New Borg El Arab, Alexandria, Egypt; 6 Center of Excellence for Drug Preclinical Studies (CE-DPS), Pharmaceutical and Fermentation Industries Development Center, City of Scientific Research and Technological Applications (SRTA-City), New Borg El Arab, Alexandria, Egypt; 7 Bio-screening and preclinical trial lab, Biochemistry Department, Faculty of Science, Alexandria University, Alexandria, Egypt; 8 Research Projects unit, Pharos University in Alexandria, Canal El Mahmoudia Street, Beside Green Plaza Complex 21648 Alexandria, Egypt

**Keywords:** COVID-19 inhibitor, Marine natural products, Machine learning, Molecular docking, Drug discovery, Biochemistry, Biotechnology, Drug discovery

## Abstract

The pressing demand for safe and efficient COVID-19 treatments has intensified interest in Natural products, especially those derived from marine organisms. In this study, a bioactive steroidal compound, 5α-cholesta-9(11)-en-3β,20β-diol, was successfully isolated from the starfish *Acanthaster planci*. Structural elucidation was achieved using HREIMS, FTIR, and advanced 1D/2D NMR spectroscopy, confirming a molecular formula of C₂₇H₄₆O₂ and characteristic functionalities including hydroxyl and double bond moieties. The compound demonstrated notable anti-SARS-CoV-2 activity, attaining 85% viral inhibition at 5 ng/µl with an IC₅₀ of 5.86 µM, as demonstrated by plaque reduction assays. Molecular docking studies demonstrated significant binding affinities toward key viral targets Mpro, NSP10, and RNA-dependent RNA polymerase with free binding energies of -26.85, -27.59, and − 35.08 kcal/mol, respectively. These affinities surpassed those of their respective co-crystallized reference ligands. In-silico ADMET profiling indicated favorable pharmacokinetic properties, including high BBB penetration, moderate intestinal absorption, and non-hepatotoxicity. Toxicity assessments predicted low carcinogenic risk, a high rat MTD, and minimal ocular and dermal irritancy. Additionally, we developed a predictive web application based on machine learning to estimate IC₅₀ values of SARS-CoV-2 inhibitors, streamlining the drug discovery process. The forecasted values nearly matched the experimental outcomes, demonstrating the model’s reliability and its potential to reduce time, cost, and risk in early-stage drug development. Moreover, machine learning models, particularly XGBoost, demonstrated excellent performance in predicting pIC₅₀ values (RMSE = 0.1357, MAE = 0.1022), supporting the development of a web-based IC₅₀ prediction application. The bioactivity prediction platform ENHPCG further validated the compound’s antiviral potential, estimating an IC₅₀ of 5.95 µM. Overall, these integrated analytical, biological, and computational approaches highlight 5α-cholesta-9(11)-en-3β,20β-diol as a potential SARS-CoV-2 inhibitor and a candidate for further pharmacological development.

**Supplementary Information:**

The online version contains supplementary material available at 10.1038/s41598-025-20443-6.

## Introduction

In the post-genomic era, computational methodologies play a pivotal role in analyzing large-scale biological data, facilitating the discovery of novel natural-product-derived drugs. A comprehensive understanding of molecular structures and chemical properties is essential for elucidating how physicochemical characteristics influence biological activity. Computer-aided drug design (CADD) encompasses various computational techniques that have significantly contributed to chemical biology and the study of structure-activity relationships (SARs)^1^. These approaches evaluate bioactivity based on ligand properties, known as chemical descriptors^2^. A key computational approach, quantitative structure–activity relationship (QSAR) modeling, is widely utilized to develop predictive models that identify critical molecular fingerprints influencing bioactivity and pharmacological properties^3^. QSAR models have been effectively utilized for diverse therapeutic targets, including antibacterial^4^, anticancer^5^, and antiviral^6^ agents. Furthermore, machine learning (ML) techniques have been leveraged to predict IC_50_ values^7^ and classify chemo-types with potential activity against viral proteases^8^.

There has been a sudden rise in viral diseases in recent years, posing significant threats to human health^9^. Natural products have been crucial in drug discovery and development during the past decades. Many bioactive compounds from plants and marine origins have been explored as effective alternatives for treating various diseases^10,11^. Sea stars are rich sources of structurally diverse natural products with broad biological activities^12^. Among these, marine steroids are particularly notable for their potential in drug development. These compounds exhibit various pharmacological activities, including anticancer, antiviral, antioxidant, antibacterial, anticoagulant, and immune-modulatory actions^9^. Marine steroids possess unique structural characteristics that contribute to their antiviral properties, either by directly inactivating virions prior to infection or by obstructing viral multiplication within host cells. Therefore, sea stars, which are rich in marine steroids, are pivotal in facilitating the identification and development of antiviral medicines.

A central challenge in antiviral drug discovery, particularly in response to emerging viral threats such as SARS-CoV-2, is the development of fast, cost-effective, and accurate approaches to screen and validate bioactive compounds. Traditional experimental methods for determining IC₅₀ values are resource-intensive and often impractical for high-throughput screening. Therefore, integrating machine learning (ML)-based prediction with experimental validation presents a promising solution. This study investigates whether a combined machine learning and experimental approach can accurately predict and validate the antiviral activity (IC₅₀) of a marine-derived steroid compound against Covid-19. To explore this, we selected *Acanthaster planci* (crown-of-thorns starfish) as a bioresource due to its rich repertoire of marine steroidal metabolites.

Echinoderms significantly impact marine ecosystems by affecting the reproductive success of benthic species and acting as primary consumers of plant matter and detritus^13^. Due to their diverse bioactive compounds, echinoderms hold significant nutritional and medicinal value, contributing to various physiological functions in humans^14^. Many echinoderm species serve as model organisms in studies related to cell biology, developmental biology, and immunology. Their defense mechanisms largely depend on a robust innate immune system, which enables them to recognize and eliminate pathogens while facilitating wound healing^13^.

Echinoderms possess a diverse array of bioactive compounds, including saponins, peptides, polysaccharides, lipids, and proteins^15^. These compounds exhibit significant physiological functions in humans, with many lacking structural or functional analogs among terrestrial secondary metabolites. Current research primarily focuses on these marine-derived secondary metabolites owing to their significant toxicological and pharmacological attributes, as well as their ecological roles within marine environments^13^. Numerous studies have explored the antimicrobial, antifungal, and antitumor actions of echinoderm-derived metabolites; however, their antiviral potential remains largely unexplored. This study investigates the antiviral properties of recently isolated bioactive substances from echinoderms, potentially providing significant insights into the antiviral immunological processes of these creatures. Among echinoderms, starfish are particularly rich in polyhydroxy steroids, steroidal glycosides (sulfated), and asterosaponins, with sulfation occurring either on the carbohydrate moiety or the steroidal structure. Sea stars primarily contain saponins and polyhydroxy steroids, which exhibit cytotoxic, hemolytic, anti-inflammatory, and antimicrobial activities^13^.

In the ongoing pursuit of effective treatments for Covid-19, accurately predicting IC₅₀ values for potential therapeutic compounds is essential for evaluating their effectiveness. Traditional experimental methods for determining IC₅₀ values are often time-consuming, costly, and hazardous. This study introduces an innovative amalgamation of machine learning approaches and experimental validation to optimize the drug discovery process. Several ML-based platforms and models have recently been proposed for predicting anti-SARS-CoV-2 compound efficacy, including deep learning-based QSAR models and virtual screening tools^3^. However, many of these approaches lack direct experimental verification, limiting their translational utility. Our study builds upon this foundation by combining a robust ML-based predictive model for estimating IC₅₀ values with experimental in vitro validation, offering a more comprehensive and practical workflow. Additionally, we introduce a user-friendly web application that allows researchers to input molecular structures and receive real-time IC₅₀ predictions. This integration of in-silico prediction with experimental confirmation enhances both the speed and reliability of early-phase antiviral drug discovery.

However, the antiviral potential of these marine steroids, especially from *Acanthaster planci*, against SARS-CoV-2 remains poorly investigated. Moreover, while machine learning (ML) techniques and cheminformatics have greatly enhanced drug screening, few studies have applied these tools to marine sterols with subsequent experimental validation. This study combines in-vitro assays, molecular docking, and ADMET analysis to evaluate an *A. planci*-derived steroid toward SARS-CoV-2. In the current research, a steroid compound was isolated and structurally characterized from *A. planci*. Its antiviral activity against Covid-19 was evaluated using in-vitro plaque reduction assays, and its molecular structure was determined through advanced spectroscopic techniques.

Concurrently, we developed a machine learning-based model to predict IC₅₀ values and performed molecular docking and ADMET study to evaluate its drug-likeness and interaction with viral targets. What distinguishes this work is the novel integration of ML-based IC₅₀ prediction with experimental validation, enabling a robust assessment of the compound’s therapeutic potential. We further developed a user-friendly web application that allows researchers to input molecular structures and receive real-time IC₅₀ predictions, an innovation aimed at reducing the cost and time associated with early-stage drug screening. This integrative strategy demonstrates a comprehensive framework for antiviral drug discovery by combining marine biotechnology with advanced computational tools. It also underscores the untapped potential of echinoderm-derived metabolites in contributing to the development of next-generation antiviral agents.

## Results

### Extraction and isolation of the compound

The process for extracting and isolating the pure compound from *Acanthaster planci* (*A. planci*) is outlined in the flowchart (Fig. S1).

The isolated compound was a white crystalline substance with the molecular formula C₂₇H₄₆O₂, indicating five degrees of unsaturation. FTIR analysis revealed bands for the hydroxyl (3423 cm⁻¹) and double bond (1737 cm⁻¹) functional groups. ¹H NMR revealed a methine proton at δ 3.55, indicating a hydroxyl at C-3. Additionally, five methyl signals were detected, supporting the structure’s sterol-like characteristics.

**Fig. 1:**
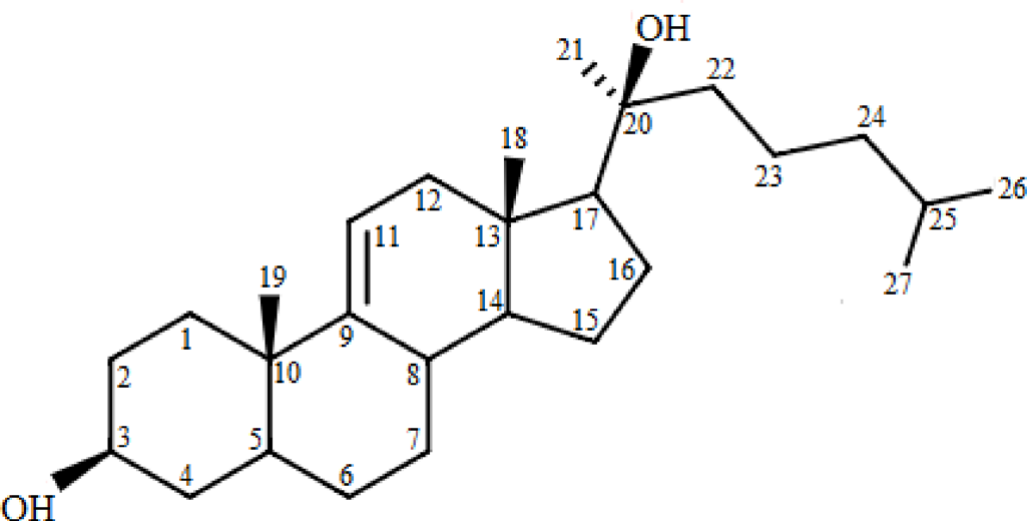
The elucidated structure of the compound obtained “5α-cholesta-9(11)-en-3β, 20β-diol”.

Carbon-13 NMR analysis revealed 27 carbon signals, including five methyl, eleven methylene, and four quaternary carbons, based on APT data. Key chemical shifts were Noted at δ 72.0 (C-3) and δ 41.7 (C-20), with methyl carbons at δ 14.1, 18.0, 27.8, 19.4, and 19.6. COSY-45°, HSQC, and HMBC spectra enabled full proton and carbon assignments. A vicinal coupling between H-11 and H-12 in the COSY-45° spectrum confirmed a ∆⁹,¹¹ double bond (Table S1, Figs. S2–S8). Based on these spectroscopic findings, the compound was identified as 5α-cholesta-9(11)-en-3β,20β-diol, as illustrated in Fig. 1.

### Anti-SARS-CoV-2 activity determination

Table 1 shows that the isolated compound inhibited SARS-CoV-2 by 85% at 5 ng/µl, with an IC₅₀ of 5.86 µM.

**Table 1:** Plaque reduction assay (SARS-CoV2).

Sample	Conc. (ng/µl)	Initial viral count	Viral count (PFU/ml)	Inhibition %
		(PFU/ml)		
The isolated Compound	5	9 × 10^5^	1.35 × 10^5^	85
	2.5		3.96 × 10^5^	56
	1.25		7.2 × 10^5^	20
	0.625		8.37 × 10^5^	7

### Molecular Docking

**Fig. 2:**
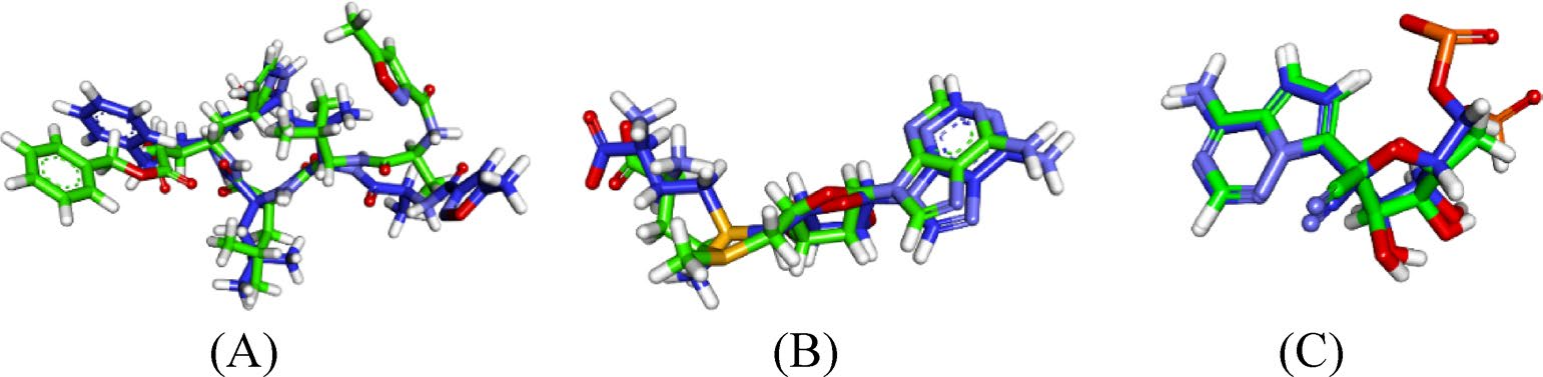
Structural alignment of co-crystallized and docked conformations for ligands: (**A**) PRD_002214, (**B**) SAM, and (**C**) F86.

To validate the docking protocol, native co-crystallized ligands were re-docked into their respective active sites. The resulting RMSD values (3.10 Å for M^pro^, 1.07 Å for nsp10, and 1.34 Å for RdRp) confirmed acceptable alignment and method reliability. These RMSD outcomes confirm the robustness and precision of the docking methodology, indicating its suitability for accurately reproducing native ligand binding modes (Fig. 2). We acknowledge the visible structural deviation between the co-crystallized ligand and the most stable re-docked pose of PRD_002214, which resulted in an RMSD of 3.10 Å. While this value slightly exceeds the commonly accepted threshold of 2.0 Å, it may be attributed to the inherent flexibility of the ligand or limitations in the docking algorithm’s conformational sampling^16^.

Comprehensive molecular docking simulations were achieved using MOE 14.0 software to explore the binding relations among the candidate compounds and selected target proteins. The calculated binding free energy (ΔG) values, summarized in Table S2, reflect the affinity and stability of these molecular complexes. The docking results indicated that the isolated compound consistently exhibited low ΔG values, suggestive of high binding affinity. The isolated compound exhibited notably strong binding affinities toward key SARS-CoV-2 targets. These binding energies were considerably lower than those of the respective co-crystallized reference ligands, highlighting the compound’s enhanced interaction potential with the viral proteins (Table S2).

### In-silico ADMET analysis

The ADMET characteristics of the isolated compound were evaluated in comparison to ritonavir as a reference, with key pharmacokinetic and toxicity properties predicted through Discovery Studio 4.0 (Table S3**)**. Remarkably, the isolated compound exhibited substantial BBB penetration, whereas ritonavir displayed limited permeability.

**Fig. 3:**
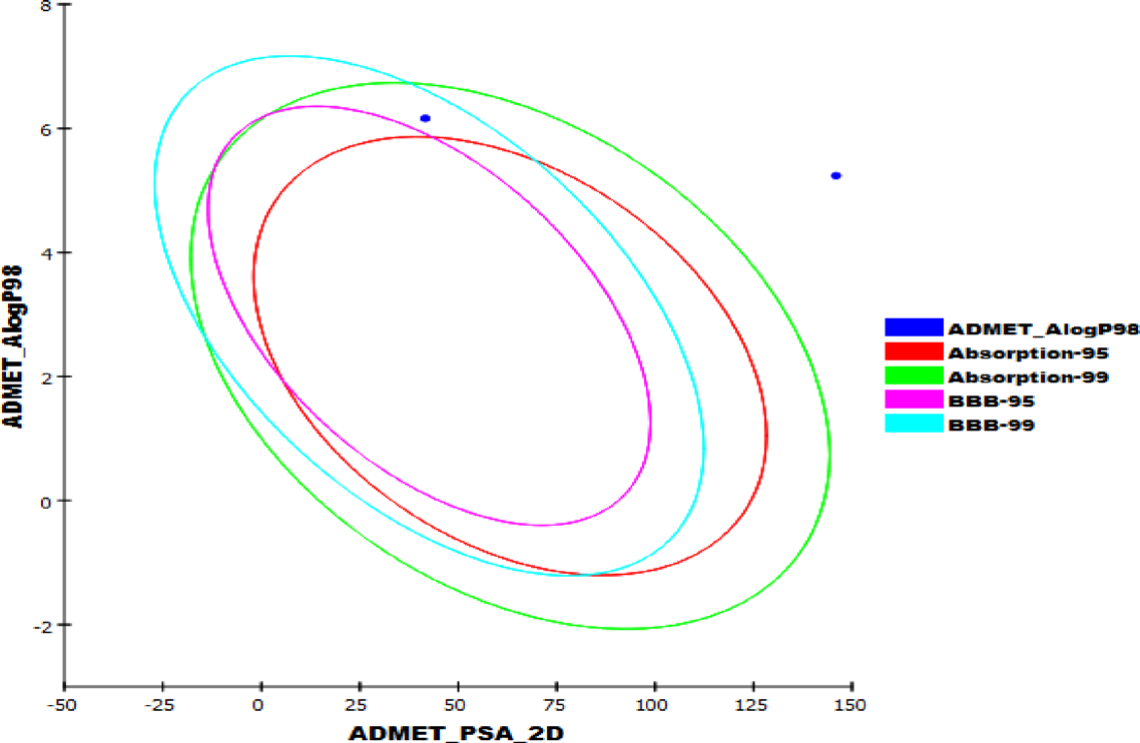
The expected ADMET profile of the isolated compound.

The isolated compound showed very high blood-brain barrier (BBB) penetration (level 0), suggesting its potential to reach the central nervous system, while ritonavir exhibited very low penetration (level 4). In terms of aqueous solubility, the isolated compound was predicted to have very low solubility (level 1), which may affect its formulation and bioavailability, although this is not uncommon for lipophilic drug candidates. Intestinal absorption was rated as moderate (level 1) for the isolated compound, slightly better than ritonavir, which had poor absorption (level 2). Importantly, the isolated compound was not predicted to inhibit the CYP2D6 enzyme, indicating a lower risk of drug-drug interactions, unlike ritonavir, which was classified as a CYP2D6 inhibitor. Moreover, the compound was Non-hepatotoxic, in contrast to ritonavir, which showed potential for Liver toxicity. Finally, the compound displayed high plasma protein binding exceeding 90%, meaning it is likely to bind strongly to blood proteins, which can influence its distribution and half-life in the body (Fig. 3).

### Toxicity studies

The toxicity assessment of the isolated compound was conducted using validated in silico predictive models implemented in Discovery Studio 4.0 software^17,18^. Key safety parameters were assessed, including FDA rodent carcinogenicity predictions, which estimated the compound’s carcinogenic potential based on its molecular structure. Carcinogenic potency (TD50) was predicted to estimate the tumorigenic dose rate at which 50% of test rodents develop tumors in chronic exposure studies^19^. The rat maximum tolerated dose (MTD) was estimated to identify the highest dose that induces no significant toxic effects^20,21^, while the oral LD50 was calculated to determine the acute lethal dose causing mortality in 50% of test animals^22^. The chronic lowest observed adverse effect level (LOAEL) was also predicted to establish the minimum dose eliciting adverse effects upon long-term exposure^23,24^. Additionally, ocular and dermal irritancy were assessed using the Draize eye test and rabbit skin irritation assay, respectively, to evaluate the compound’s potential to cause eye and skin irritation^25^.

The in-silico toxicity assessment revealed that the isolated compound exhibited a favorable safety profile relative to Ritonavir. It was anticipated to be non-carcinogenic and showed a higher TD50 (2.651 mg/kg/day) and MTD (0.047 g/kg). The compound also demonstrated a significantly higher oral LD50 (2.855 mg/kg/day) than Ritonavir (0.477 mg/kg/day). Although its LOAEL (0.005 g/kg) was lower than Ritonavir’s. These comprehensive toxicity predictions provide essential insights into the compound’s safety, supporting its potential applicability in further pharmacological and biomedical research.

### Machine learning modeling

This section evaluates the prediction efficacy of machine learning models for pIC_50_ estimation. The overall accuracy of different models following hyperparameter tuning is summarized in Table 2, while Table 3 presents the optimal parameters identified through the random search method. Among the evaluated models, the XGB algorithm demonstrated superior predictive performance, yielding the lowest root mean square error (RMSE) and mean absolute error (MAE) across both training and testing datasets (Table 2).

**Fig. 4:**
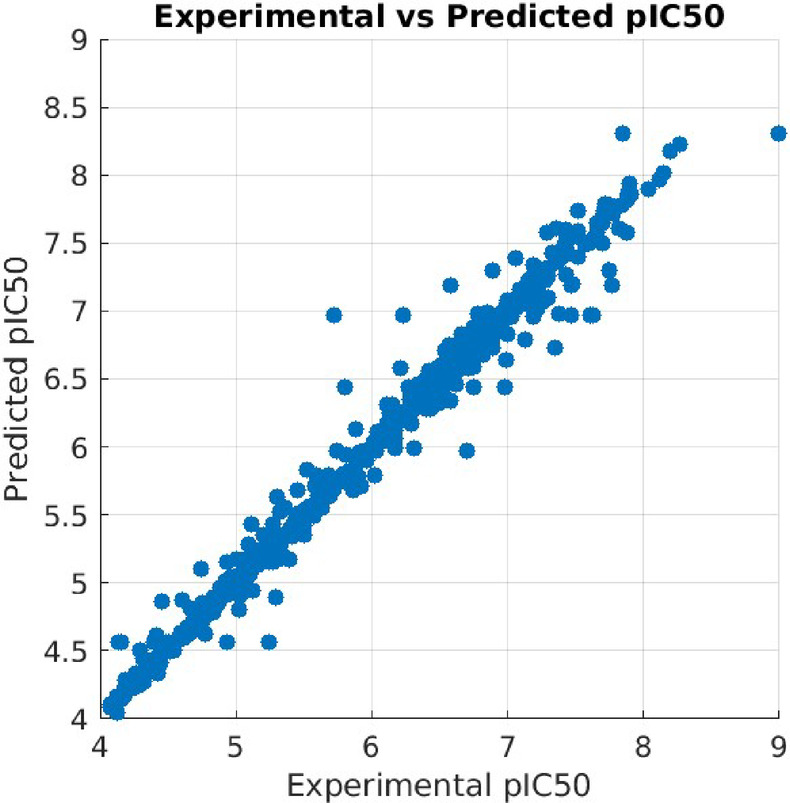
Comparison of experimental and predicted pIC50 values generated by the machine learning model utilizing MACCS fingerprint descriptors.

Accordingly, this model was selected for the deployment of the IC_50_ prediction web application, utilizing the optimized parameters listed in Table 3. Furthermore, Fig. 4 illustrates the relationship between experimentally determined and predicted pIC₅₀ values generated by the model employing MACCS (Molecular ACCess System) fingerprint-based descriptors.

**Table 2:** Summary of the predictive performance of machine learning models for estimating the pIC₅₀ values against SARS-CoV-2.

ML model	Training		Testing	
	RMSE	MAE	RMSE	MAE
XGB	**0.0370**	**0.0202**	**0.13574**	**0.10219**
RF	0.0547	0.0392	0.14095	0.10255
SVR	0.0407	0.0272	0.7605	0.5578

**Table 3:** Best parameters of ML models for predicting pIC_50_ of SARS-CoV-2.

Model	Best parameters
**XGB**	Colsample_bytree: 0.8954454315573364 Learning_rate: 0.07200530796302361 Max_depth: 8 N_estimators: 496 Reg_alpha: 0.21458911970837413 Reg_lambda: 0.37725908322797586 Subsample: 0.71168893969307
**RF**	bootstrap: True max_depth: 44 min_samples_leaf: 1 min_samples_split: 2 n_estimators: 355
**SVR**	C: 546.8102793432797 degree: 3 epsilon: 0.025656646227779294 kernel: poly

### Roadmap for novel compounds in predicting IC_50_ for SARS-CoV-2

To facilitate the prediction of IC_50_ values for SARS-CoV-2, we employed DECIMER (Deep Learning for Chemical Image Recognition)^26^, an advanced AI-driven tool designed for the automated extraction of chemical structures from images. This web-based platform utilizes deep learning algorithms to accurately recognize and convert chemical structure depictions into machine-readable formats such as SMILES. By streamlining the digitization of chemical information from research articles, patents, and other image-based sources, DECIMER enhances the efficiency of cheminformatics workflows.

**Fig. 5:**
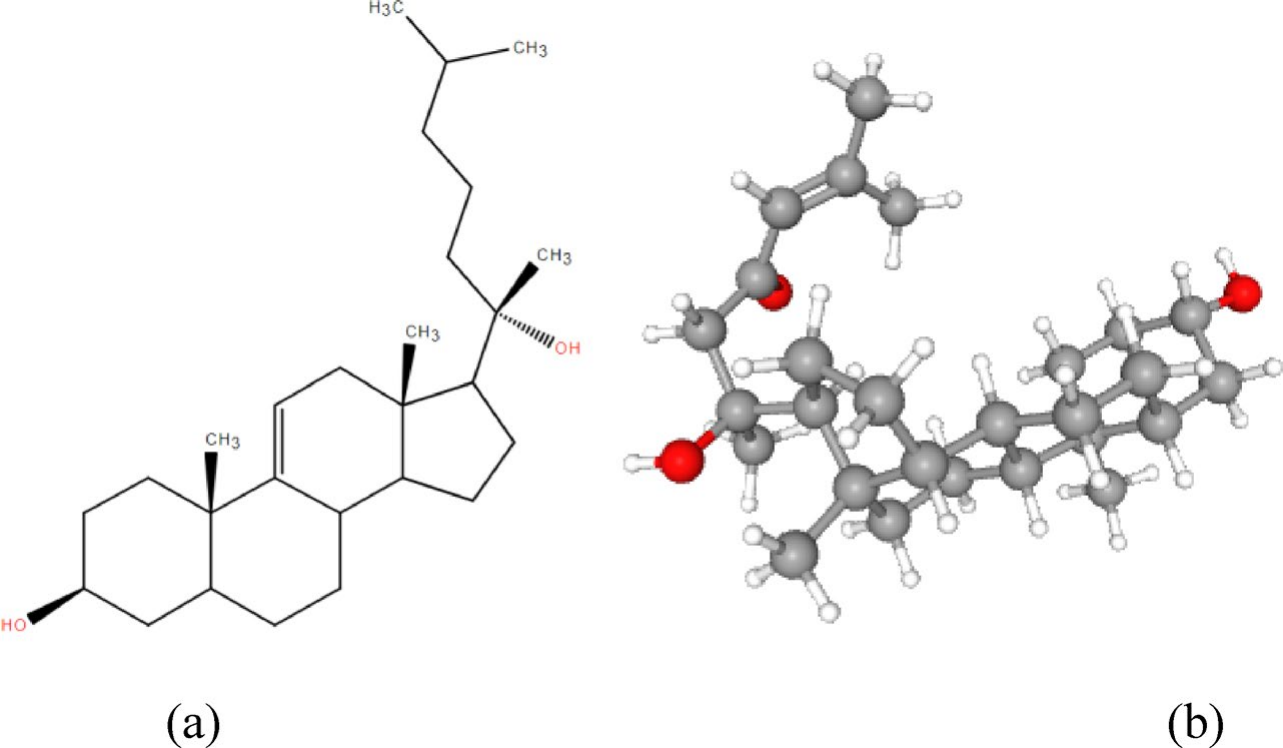
**(a)** 2 d and **(b)** 3 d structure of the isolated compound.

In our study, we uploaded the 2D structural representation of the target compound (Fig. 5a) onto the DECIMER platform. The tool successfully generated the corresponding 3D molecular structure (Fig. 5b) and provided its SMILES notation, enabling further computational analysis and predictive modeling. The SMILES notation for the compound is as follows:

CC(C)CCC[C@@](C)(C1CCC2C3CCC4C[C@H](CC[C@]4(C)C3 = CC[C@@]21 C)O)O

This representation encodes the molecular structure in a linear, machine-readable format, facilitating computational modeling and cheminformatics analyses.

**Fig. 6:**
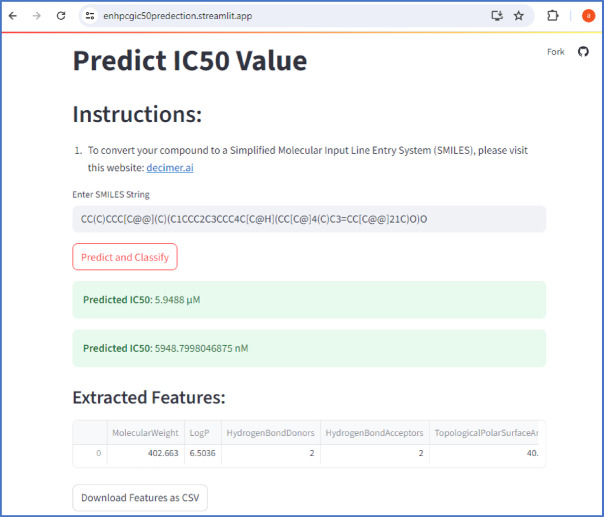
The Predicted IC_50_ value for the isolated compound using ENHPCG bioactivity service.

The IC₅₀ value of the isolated compound toward SARS-CoV-2 M^pro^ was predicted using the bioactivity prediction module integrated within the ENHPCG platform. Through the proposed web-based application for bioactivity assessment (https://enhpcgic50predection.streamlit.app/), the compound yielded a predicted IC₅₀ value of 5.9488 µM, as illustrated in Fig. 6.

### Validation of the proposed XGB model for IC_50_ prediction in SARS-CoV-2

To assess the predictive performance of our proposed XGB model, we conducted a validation study by comparing its IC_50_ predictions with experimentally determined values for 32 bicycloproline derivatives targeting SARS-CoV-2 main protease (Mpro). Table S4 presents a comparative analysis between the experimental IC_50_ values reported by^27^ and the predicted values by our machine learning model. The results indicate a strong correlation between the predicted and experimental data, underscoring the accuracy and reliability of our approach in estimating the bioactivity of potential SARS-CoV-2 inhibitors.

To evaluate the predictive accuracy of our XGB model for lower-activity compounds against Mpro, we compared the experimentally determined IC_50_ values reported by^28^ with the predicted IC_50_ values for selected compounds targeting the Mpro of SARS-CoV-2 (Table S5). The findings indicate that the model effectively predicts IC_50_ values with reasonable accuracy, demonstrating a strong correlation with the experimental data.

**Fig. 7:**
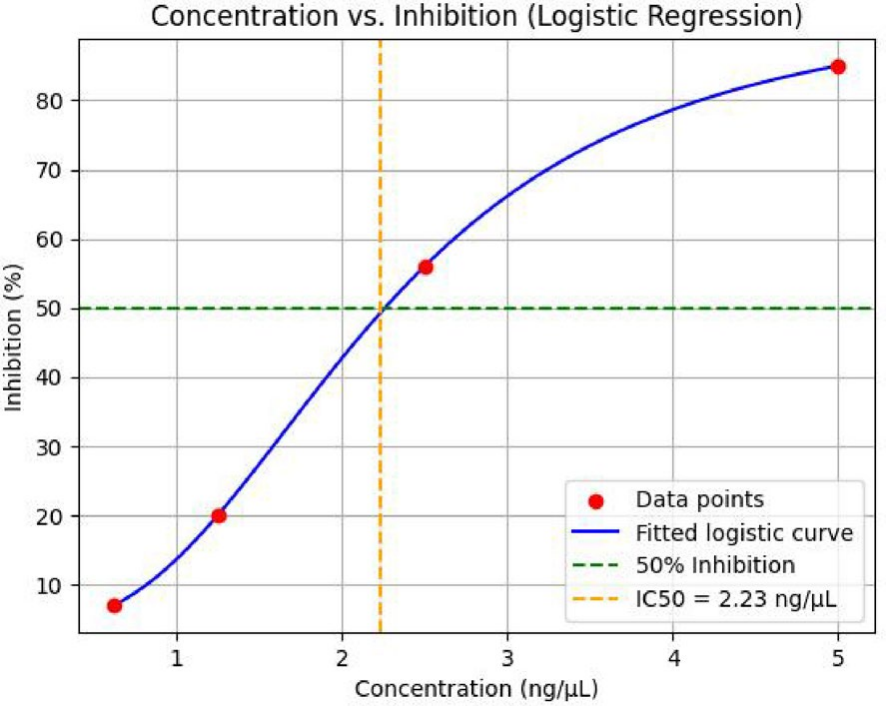
Dose-Response Curve to calculate IC_50_ experimentally.

For our isolated compound, experimental IC₅₀ determination was carried out using the logistic regression method, as shown in Fig. 7. The experimentally derived IC₅₀ was 2.23 ng/µL. Using the RDKit cheminformatics library, we computed the molecular weight (402.66 g/mol) and converted this value into µM units to enable direct comparison with model predictions as follows:

$$\:IC50\left(\mu\:M\right)=\frac{IC50\left(ng/\mu\:l\right)}{Molecular\:Weight\left(g/mol\right)}\times\:{10}^{3}=\:\frac{2.23\:}{402.66}\times\:{10}^{3}=5.5469\:{\upmu\:}\text{M}$$

This case study demonstrates how combining experimental assays with machine learning predictions provides a robust, dual-layer validation approach that strengthens confidence in candidate compound selection.

## Discussion

In this study, we isolate a novel compound from the *Acanthaster planci* starfish with potential inhibition of COVID-19 infection. Comprehensive structural characterization, molecular docking analyses, and in-silico ADMET evaluations were achieved. Our primary focus is on developing a robust model for predicting the IC_50_ values of compounds with activity against SARS-CoV-2. To improve accessibility, we have also developed a web application that enables researchers to input compounds and efficiently obtain predicted IC_50_ values. The model’s validity was assessed by comparing the predicted IC_50_ values with experimental measurements obtained through in-vitro bioassays, demonstrating its effectiveness in reducing both the cost and risk associated with IC_50_ determination.

The urgent demand for a safe and effective drug to combat COVID-19 has intensified research into natural products, which have long served as fundamental components of traditional medicine. These bioactive compounds represent a key resource for developing novel therapeutic agents, making their exploration a crucial step in drug discovery. In the treatment of retroviral infections, several molecular targets can be inhibited.

In-silico target identification offers a rapid, cost-effective method for screening natural products. Key computational tools include QSAR modeling and molecular docking.

Docking helps reveal interactions between natural compounds and target biomacromolecules, aiding mechanism-of-action studies. This strategy supports drug discovery and has been central to developing natural product-based therapeutics.

**Fig. 8:**
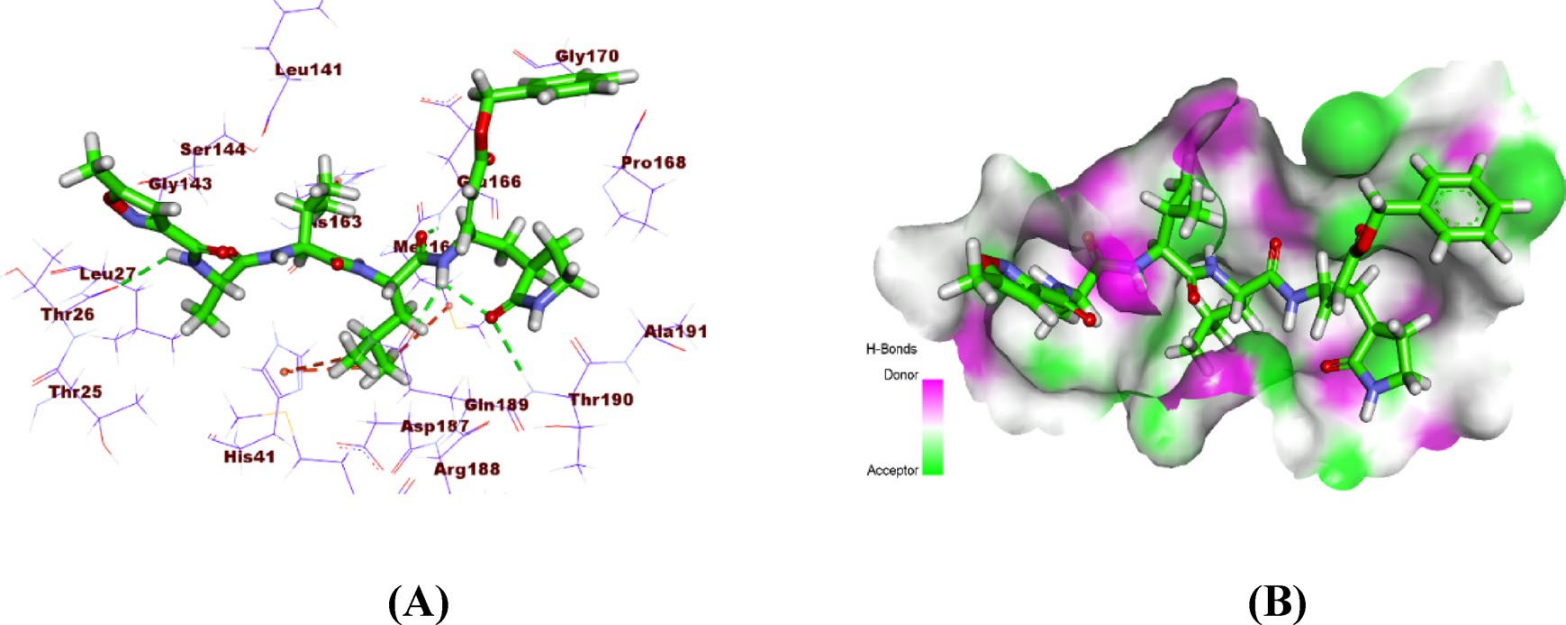
**A)** 3D docking and **B**) Surface mapping of the co-crystallized ligand (PRD_002214) within the active site of the COVID-19 main protease.

The Mpro protein active site contains four distinct pockets, each interacting with specific ligand moieties. The co-crystallized ligand (PRD_002214) forms four hydrogen bonds and three hydrophobic interactions. Key interactions include hydrogen bonds with Thr190, Gln189, and Thr26, and hydrophobic contacts with His41 and Met165. These interactions collectively stabilize the ligand within the active site (Fig. 8).

**Fig. 9:**
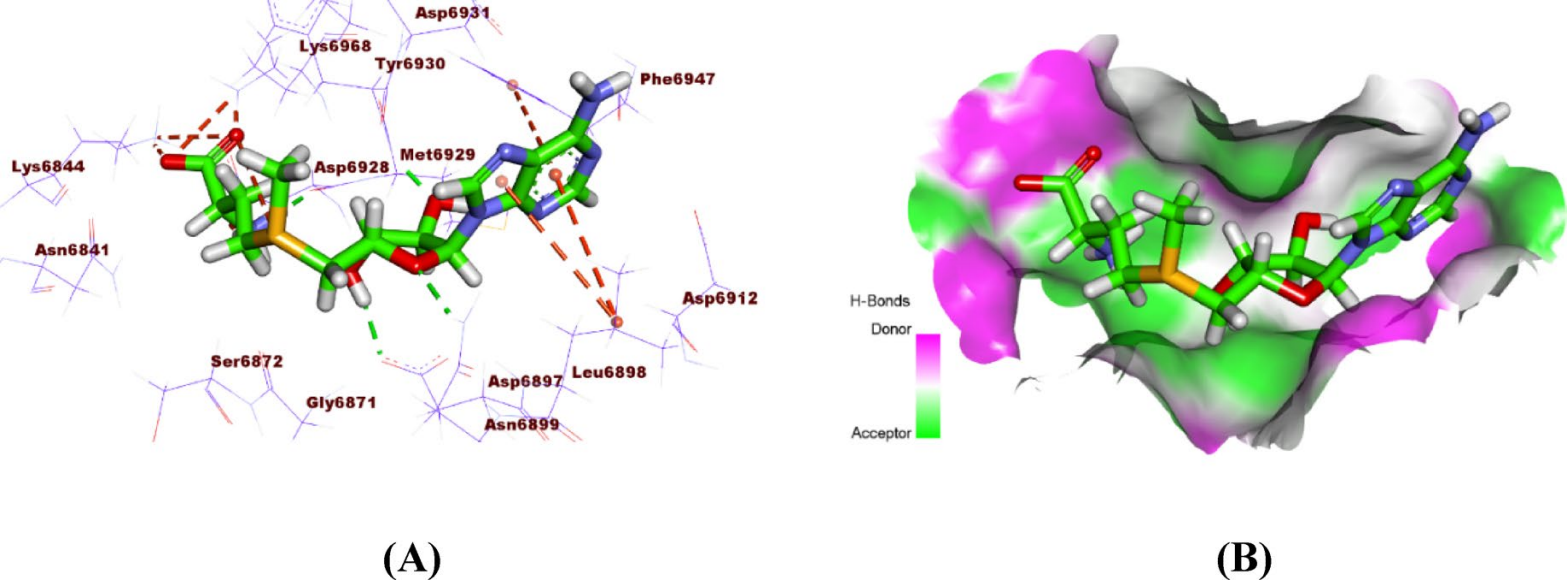
**(A)** 3D structure, and **(B)** surface mapping of SAM bound to the active site of SARS-CoV-2 NSP10.

The co-crystallized ligand SAM interacts with SARS-CoV-2 nsp10 through three hydrogen bonds, along with several hydrophobic and electrostatic interactions, thereby guaranteeing stable occupancy of the active site. The critical residues implicated are Asn6899, Tyr6930, Asp6928, Phe6947, and Leu6898 (Fig. 9).

**Fig. 10:**
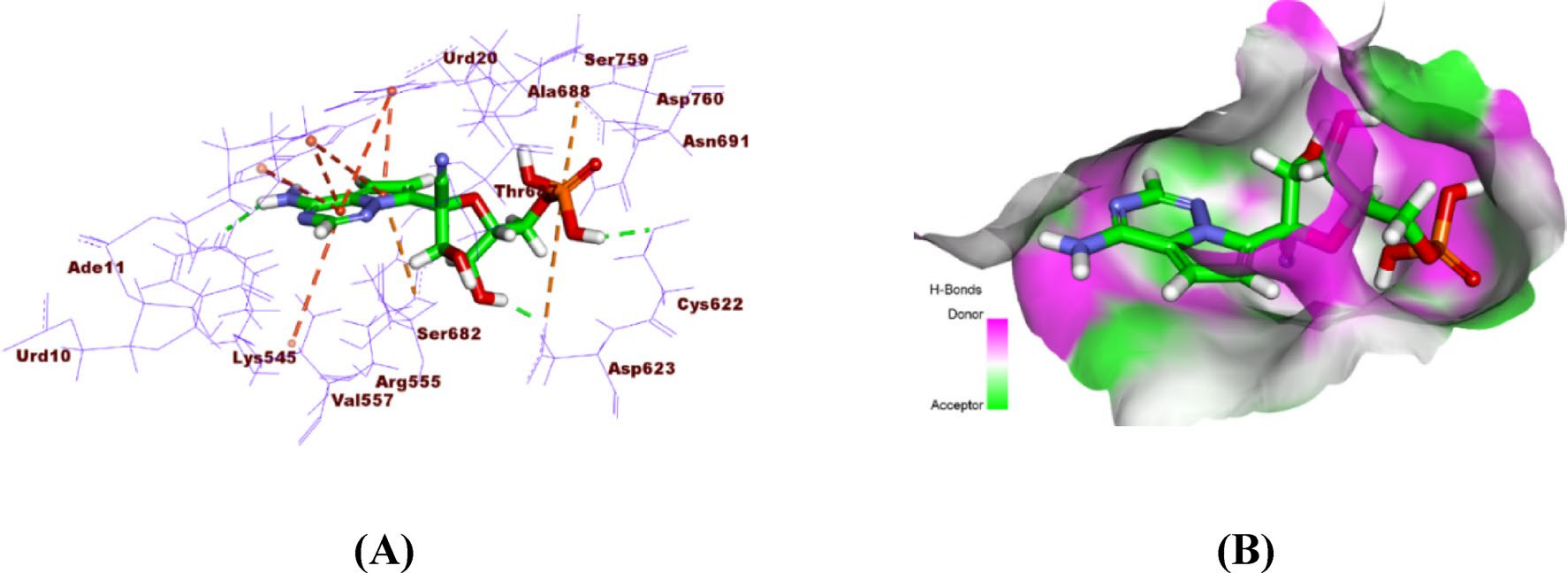
**A**) 3D binding, and **B)** surface mapping of the co-crystallized ligand F86 within the active site of COVID-19 RdRp.

The co-crystallized ligand F86 binds firmly to the active site of SARS-CoV-2 RdRp via many interactions. This comprises three hydrogen bonds, six hydrophobic relations, and two electrostatic contacts. The critical residues implicated are Arg555, Val557, Asp623, Asp760, and Cys622. This interaction network improves the ligand’s binding affinity and conformational stability (Fig. 10).

**Fig. 11:**
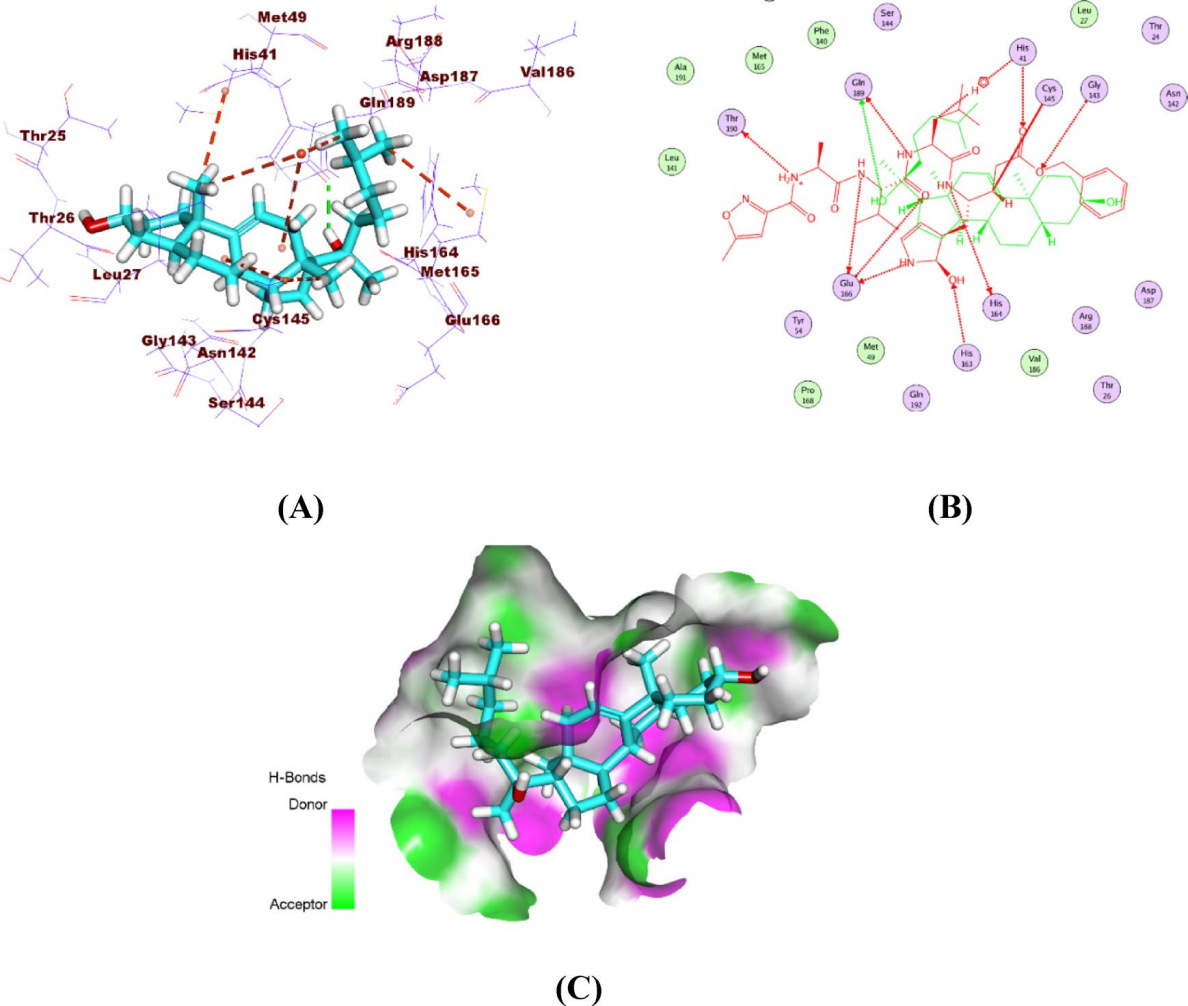
Binding of the isolated compound to the SARS-CoV-2 main protease: (**A**) 3D binding pose, (**B**) 2D overlay with the co-crystallized ligand, and (**C**) surface mapping within the active pocket.

The isolated compound bound to three key pockets of SARS-CoV-2 Mpro, forming seven hydrophobic contacts and one hydrogen bond. Its binding orientation closely resembled that of the co-crystallized ligand, indicating similar active site interactions. The (1R,4R)−4-methylcyclohexan-1-ol and indene moieties interacted with Met49, His41, and Cys145 across the first two pockets. The side chain reached the third pocket, producing a hydrogen bond with Gln189 and hydrophobic contacts with Met165 and His41. (Fig. 11).

**Fig. 12:**
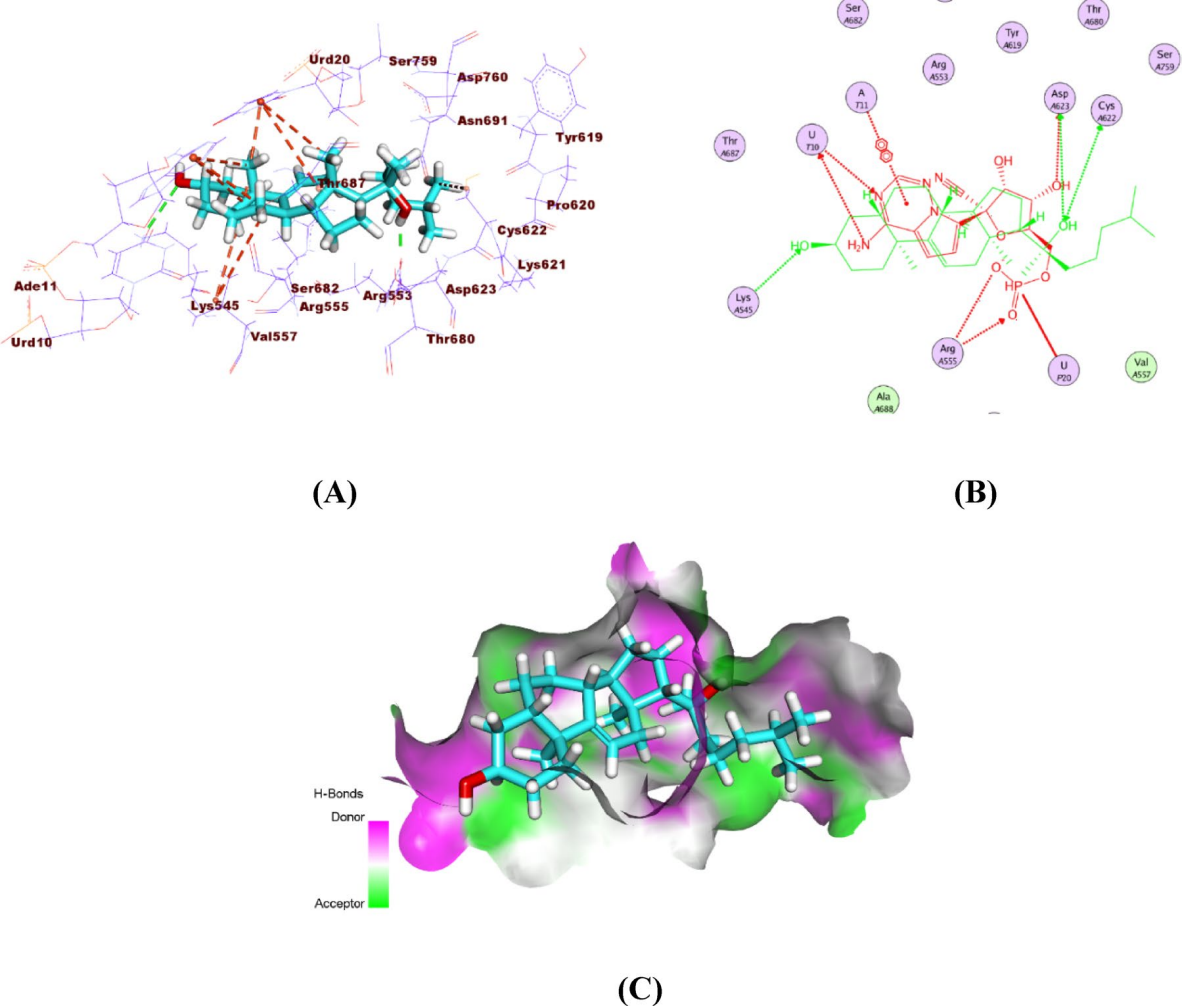
Binding interaction of the isolated compound with SARS-CoV-2 RdRp, showing **(A)** 3D, **(B)** 2D, and **(C)** surface views. The compound aligns with the co-crystallized ligand, confirming its fit within the enzyme’s active site.

The isolated compound exhibits stable binding within the NSP10 active site via four hydrophobic connections and one hydrogen bond. The (1r,4r)−4-methylcyclohexan-1-ol moiety forms a hydrogen bond with Asp6912 and hydrophobic contacts with Leu6898 and Phe6947. Additional hydrophobic interactions with Pro6932 from two separate moieties further stabilize the ligand (Fig. S9). The isolated compound showed strong binding with RdRp, forming ten hydrophobic relations and two hydrogen bonds within the active site. Key fragments interacted with residues such as Lys545, Val557, and Urd20, enhancing binding stability. Additionally, the side chain formed a hydrogen bond with Asp623, further supporting its inhibitory potential (Fig. 12).

A detailed analysis of the docking results revealed that only specific functional groups within the isolated compound actively participate in key interactions with the target binding site. The steroidal core structure of the compound establishes hydrophobic contacts with non-polar residues lining the binding pocket, contributing to the stabilization of the ligand within the active site. Additionally, the presence of hydroxyl groups enables hydrogen bonding interactions with polar residues such as Ser144 and His163, which are known to be critical for ligand recognition in the main protease active site. These polar interactions contribute substantially to the improvement of binding affinity and specificity. Furthermore, the spatial orientation of the functional groups dictates the ligand’s ability to fit properly within the pocket, influencing the overall docking score and binding mode. This selective engagement of chemical moieties underscores the importance of molecular complementarity in structure-based drug design and supports the observed docking performance of the isolated compound.

As presented in Table S6, in-silico toxicity assessments indicated that the isolated compound exhibited low toxicity across multiple predictive models. It was classified as non-carcinogenic according to the FDA rodent carcinogenicity model. The compound displayed a higher TD₅₀ value (2.651 mg/kg/day) and a greater predicted maximum tolerated dose (0.047 g/kg) compared to ritonavir (1.369 mg/kg/day and 0.038 g/kg, respectively), indicating a favorable toxicity profile.

Furthermore, the compound demonstrated an oral LD₅₀ of 2.855 mg/kg body weight/day, significantly exceeding that of ritonavir (0.477 mg/kg body weight/day), indicating a lower potential for acute toxicity. The lowest observed adverse effect level (LOAEL) was identified as 0.005 g/kg body weight, which is lower than ritonavir’s LOAEL of 0.014 g/kg body weight, suggesting enhanced safety at low-dose exposure. Additionally, predictive models identified the compound as a potential ocular irritant while showing no skin irritation potential, reinforcing its favorable safety characteristics.

The comparison between the isolated steroid and known antiviral agents, such as ritonavir, underscores its therapeutic potential. Notably, the compound exhibits favorable binding affinity, suggesting a strong interaction with the target viral protein, which is essential for effective inhibition. This computational prediction is further supported by molecular docking and dynamics studies, positioning the compound as a viable antiviral candidate. Additionally, its ADMET profile reveals lower acute toxicity (higher LD50) and a higher maximum tolerated dose (MTD) compared to ritonavir, indicating that it may be better tolerated in-vivo. These pharmacokinetic advantages, combined with predicted non-carcinogenicity and skin safety, enhance its attractiveness for further development.

What distinguishes the isolated compound most significantly is its unique chemical structure and biological origin. As a novel marine-derived steroid, it represents a new chemotype not commonly found among current antiviral drugs, which are often synthetic or peptide-based. This structural novelty may reduce the risk of cross-resistance with existing therapies and provide new mechanisms of action. Moreover, the compound’s balanced profile of efficacy (via binding affinity) and safety (via in-silico toxicity studies) suggests a promising starting point for lead optimization and preclinical investigation. Its potential as a safe and effective antiviral agent merits further validation through in-vitro assays and mechanistic studies.

For the majority of compounds analyzed, the predicted IC₅₀ values exhibited a strong correlation with experimentally determined values, underscoring the model’s ability to capture key molecular determinants of antiviral activity. This correlation suggests that the machine learning approach, particularly the XGB model, is well-suited for early-stage screening and prioritization of SARS-CoV-2 inhibitors. For example, compounds such as Ebselen, Disulfiram, and Tideglusib were predicted to have low IC₅₀ values, consistent with previously reported experimental data supporting their inhibitory potential.

Nonetheless, certain discrepancies were observed. In cases like Carmofur and PX-12, the model tended to overestimate the IC₅₀ values, potentially due to factors such as off-target effects, conformational flexibility, or specific interactions not captured by the chosen molecular descriptors. These deviations highlight a key limitation of the current model: it relies solely on the MACCS fingerprint, which, while efficient and interpretable, may not fully represent complex bioactive features or three-dimensional interactions. Furthermore, the training dataset size, though curated and specific, may limit generalizability to structurally novel compounds or those with underrepresented scaffolds.

Despite these constraints, the model demonstrates substantial utility as a computational filter to reduce experimental workload and resource consumption. This is particularly beneficial when focusing on highly active compounds (e.g., IC₅₀ < 1 µM), where early prediction can direct attention to the most promising leads, as summarized in Table S4.

## Conclusion

This study successfully isolated and characterized a steroid compound from starfish, demonstrating its potential as a COVID-19 inhibitor. Using a combined analytical approach, we identified the structural and functional properties of the compound, which exhibited promising in-vitro antiviral activity. Furthermore, machine learning models provided predictive insights into the compound’s inhibitory potential, reinforcing its relevance as a candidate for further drug development.

Our findings highlight the significance of marine-derived natural products in antiviral research and underscore the power of integrating computational and experimental methodologies for drug discovery. Future studies should focus on in-vivo evaluations and structural modifications to enhance the compound’s efficacy and pharmacokinetic properties, paving the way for novel marine-based therapeutics against COVID-19 and other viral infections.

This study exposed that *A. planci* is a promising reservoir of bioactive compounds with significant potential for the development of new therapeutic agents. The development of our predictive model for IC_50_ values has proven effective in estimating the potency of compounds against SARS-CoV-2, with validation against experimental data confirming its accuracy. The integrated web application enhances accessibility, allowing researchers to efficiently obtain predictions and streamline the drug discovery process.

This model significantly reduces the cost and risk associated with traditional IC_50_ measurements. The strong correlation observed between predicted and experimental IC₅₀ values affirms the model’s applicability in real-world drug screening scenarios. This serves as a foundation for incorporating ML-driven prediction into antiviral discovery pipelines. Future investigations will aim to enhance the model’s predictive performance by integrating expanded datasets and employing more sophisticated machine learning algorithms.

Further validation with a broader range of experimental datasets is planned to enhance the model’s robustness. Additionally, we aim to expand the web application’s features to include predictive capabilities for other viral targets and integrate it with existing drug discovery pipelines. This study represents a preliminary exploration of the antiviral potential of the isolated compound. While the selection of the three targets was guided by existing literature and their known relevance to SARS-CoV-2, we acknowledge the limitation of not employing computational target prediction tools such as PharmMapper or SEA. In future work, we intend to incorporate such in silico target fishing approaches to ensure a more comprehensive and unbiased identification of potential molecular targets.

## Methods

### Sample collection

Live *Acanthaster planci* specimens (100–300 g) were collected in July 2022 from the Red Sea near Hurghada, Egypt (27.28°N, 33.77°E), at a depth of 4–10 m. Identification was based on morphological traits. Specimens were washed, sealed in bags, frozen, and transported under refrigeration. Samples were stored at − 20 °C for later analysis. Taxonomic classification was confirmed according to the World Register of Marine Species. The starfish belongs to Phylum Echinodermata, Class Asteroidea, Order Valvatida, Family Acanthasteridae. All procedures complied with NIOF ethical standards and ARRIVE guidelines for marine invertebrate research. All methods were performed in accordance with the relevant guidelines and regulations. All the experimental protocols were approved by the Institutional Animal Care and Use Committee (IACUC), National Institute of Oceanography and Fisheries.

### Extraction and isolation

Whole-body tissue of *A. planci* (1.5 kg) was extracted using chloroform/methanol at room temperature, repeated three times. The pooled extracts were dried under reduced pressure and stored at − 20 °C. Crude extract underwent purification using silica gel column chromatography.

A stepwise elution gradient from hexane and dichloromethane to methanol was applied for fractionation (Fig. S1).

### Spectroscopic characterization of the isolated compound

The structural elucidation of the isolated compound was accomplished through a comprehensive set of spectroscopic techniques, including NMR, FT-IR, and mass spectrometry.

### NMR spectral analysis

Proton (^1^H) and carbon (^13^C) nuclear magnetic resonance (NMR) spectra were obtained using a Bruker AVANCE 400 MHz spectrometer at the University of Winnipeg, Canada, with deuterated chloroform (CDCl₃) as the solvent. Structural elucidation was further supported by two-dimensional NMR techniques, including COSY, HSQC, HMBC, and NOESY. Chemical shifts (δ) are reported in ppm, and coupling constants (J) are given in Hz.

### FT-IR analysis

FT-IR spectroscopy was utilized to determine the functional groups within the isolated compound. The sample was finely ground and uniformly blended with an equal amount of dried KBr, then pressed into a pellet. Spectral data were acquired in transmission mode using a Bruker Alpha FT-IR spectrometer, spanning the wavenumber range of 4000 to 400 cm⁻¹.

### GC-MS analysis

GC-MS analysis was performed to recognize and verify the structure of the isolated bioactive molecule. A high-resolution GC-MS system, the Agilent 7890 A-5975 C, was employed, utilizing helium as the carrier gas. The temperature gradient was set from 90 °C to 300 °C. Compound identification relied on spectral matching with known reference databases through automated techniques.

### Assessment of Anti-SARS-CoV-2 activity

The antiviral activity of the isolated compound toward SARS-CoV-2 was assessed through a plaque reduction assay, performed according to the methodology outlined by^29^. This experiment was carried out at the central laboratories of the Chemical Warfare Department in Egypt. The compound’s efficacy was determined by calculating the percentage reduction in viral plaque formation relative to untreated control wells, providing a measure of its antiviral potential as follows:

$$\:\%\:inhibition=\:\frac{viral\:count\:\left(untreated\right)-viral\:count\:\left(treated\right)}{viral\:count\:\left(untreated\right)}\:\times 100$$

### Molecular docking studies

The three-dimensional crystal structures of the selected target proteins: (i) COVID-19 main protease (M^pro^) (PDB ID:6lu7, resolution: 2.16 Å), (ii) non-structural protein (nsp10) (PDB ID: 6W4H, resolution: 1.80 Å), and (iii) RNA-dependent RNA polymerase (PDB ID: 7BV2, resolution: 2.50Å) were retrieved from the Protein Data Bank (http://www.pdb.org). Molecular docking was performed using the Molecular Operating Environment (MOE) software platform^30^ to evaluate the binding affinity and interaction profiles of the test compounds with the active sites of the target proteins.

Prior to docking, water molecules were removed from the protein structures, and a single, functionally relevant protein chain was retained for each target. The co-crystallized ligands served as reference molecules for validation of the docking protocol. The protein structures were protonated and energy-minimized using the MMFF94x force field after hiding hydrogen atoms. Binding sites were identified and defined based on previously reported methodologies^31–33^, enabling accurate docking simulations and comparative analysis of binding energies and poses.

The chemical structures of the investigated compound and the co-crystallized Ligands were initially constructed using ChemBioDraw Ultra 14.0 and exported in Structure Data File (SDF) format. These files were subsequently imported into Molecular Operating Environment (MOE) software, where the 3D structures were protonated, and energy minimization was performed using the MMFF94x force field.

To validate the docking protocol, redocking of each co-crystallized ligand into its corresponding binding site was conducted. Successful validation was indicated by low root-mean-square deviation (RMSD) values between the docked poses and the original crystallographic conformations, following established methodologies with acceptable cutoff: RMSD ≤ 2.0 Å^34,35^. Docking simulations were executed using the default settings in MOE, generating 30 docking poses per Ligand via genetic algorithm-based conformational searches. The docked complexes were subsequently examined and visualized via Discovery Studio 4.0 software^35–39^. Figure (S10) indicate a schematic diagram for the docking workflow.

### In-Silico ADMET prediction

The compound was structurally optimized using the CHARMM force field, then analyzed using the ADMET Descriptors protocol in Discovery Studio 4.0. This evaluation predicted its pharmacokinetic and toxicity-related properties^34–37^.

### In-Silico toxicity assessment

Toxicological profiling of the compound was also conducted using Discovery Studio 4.0. Ritonavir served as the reference control drug^40^. Ritonavir was selected as a reference compound due to its well-established antiviral activity and its reported inhibitory effects on SARS-CoV-2 main protease in several in-silico and in-vitro studies. It has been widely used in molecular docking and ADMET studies as a benchmark for evaluating the pharmacokinetic and toxicity profiles of potential antiviral candidates. After structural optimization using the CHARMM force field, toxicity endpoints were evaluated via the Toxicity Prediction (Extensible) protocol. Multiple predictive parameters were evaluated to determine the compound’s safety profile^41–43^.

### Machine learning model development

A dataset comprising 758 small-molecule inhibitors of the SARS-CoV-2 main protease (Mpro) was obtained from a publicly available and curated dataset^3^. The compounds span a broad range of chemical scaffolds and activity values, making them suitable for training robust regression models for IC₅₀ prediction. To represent each molecule’s structural and physicochemical features, we computed molecular fingerprints, which are widely used in QSAR modeling to encode relevant chemical information. Among the various fingerprint types available, such as PubChem, ECFP, CDK, Substructure, and MACCS, we selected the 166-bit MACCS (Molecular Access System) keys, generated using the RDKit cheminformatics library.

MACCS fingerprints were chosen for several reasons. First, they offer broad coverage of substructural features, enabling an informative representation of bioactivity-relevant chemical motifs^44^. Second, their low dimensionality helps mitigate the risk of overfitting and enhances model interpretability, particularly in datasets of limited size. Third, they are computationally efficient, making them well-suited for large-scale virtual screening and seamless integration into web-based predictive platforms. Although more complex and higher-dimensional fingerprints, such as Klekota-Roth or ECFP, are available and commonly used in cheminformatics applications, preliminary experiments revealed that these alternatives provided only marginal improvements in predictive performance while significantly increasing computational overhead. Given this tradeoff, MACCS fingerprints were deemed appropriate and optimal for the goals of the current study.

### Feature selection and model training

The MACCS fingerprints served as input features for model training. No additional dimensionality reduction or filtering was applied due to the relatively low feature count (166). The dataset was randomly split into 80% training and 20% testing subsets. To validate model generalization and prevent overfitting, we employed 4-fold cross-validation during hyperparameter tuning. Three regression algorithms were evaluated: Random Forest (RF), Extreme Gradient Boosting (XGB), and Support Vector Regression (SVR). Performance was assessed using standard regression metrics (e.g., RMSE, MAE).

Subsequently, several widely adopted regression algorithms were trained and evaluated, including Extreme Gradient Boosting Regressor (XGB), Random Forest (RF), and Support Vector Regression (SVR). These models were assessed for their predictive performance on the prepared dataset. The XGB model, recognized for its computational efficiency and high predictive accuracy when handling large-scale datasets, was evaluated for its capability to address real-world regression tasks using optimized resource utilization^45^. The RF model, which constructs an ensemble of decision trees to enhance predictive robustness and reduce variance, was tested for its reliability and generalization performance^3^. SVR, known for its effectiveness in managing high-dimensional feature spaces and its flexibility in non-linear regression, was also examined for its applicability to the current dataset^46^.

Model performance was quantitatively assessed using standard regression evaluation metrics to determine suitability for the intended QSAR application. The biological activity values (IC₅₀, in µM) were transformed into their logarithmic form (pIC₅₀), where pIC₅₀ = -log₁₀(IC₅₀), a commonly used transformation in QSAR studies to normalize the data and improve model interpretability^47^. Hyperparameter optimization for each model was performed using MATLAB’s fitrensemble function, integrated with a custom random search strategy, evaluating combinations across predefined hyperparameter ranges. This ensured robust exploration of the hyperparameter space.

### Computational framework and model optimization

All analyses were conducted on the Egyptian National High-Performance Computing Grid (ENHPCG)^48^, using MATLAB R2022a Update 4. The grid offers scalable computing infrastructure to handle large datasets and intensive training processes^49^. To optimize model performance, we utilized MATLAB’s *fitrensemble* function, integrated with a custom random search algorithm for hyperparameter tuning. This method involved specifying predefined ranges for each hyperparameter and systematically evaluating various combinations to identify those yielding the highest predictive accuracy. Hyperparameter optimization was performed using a 4-fold cross-validation strategy to ensure robustness and generalizability in predicting pIC₅₀ activity. The optimal hyperparameter configurations for each regression model were as follows:

Random Forest (RF): *max_depth*, *min_samples_leaf*, *min_samples_split*, and *n_estimators*.

Extreme Gradient Boosting (XGB): *colsample_bytree*, *learning_rate*, *max_depth*, *n_estimators*, along with regularization parameters *reg_alpha*, *reg_lambda*, and *subsample*.

Support Vector Regression (SVR): *C*, *degree*, *epsilon*, and a polynomial (*poly*) kernel.

By combining *fitrensemble* with the randomized search approach, we aimed to maximize the predictive performance and efficiency of the regression models in forecasting pIC₅₀ values.

### Image-to-SMILES conversion with DECIMER

Several bioactive compounds from recent literature were available only in image (2D structure) format, without associated SMILES strings. To incorporate these into our dataset and prediction pipeline, we used DECIMER, a deep-learning-based tool for image-to-SMILES conversion. This integration was essential for digitizing chemical structures from scientific figures and enabling their use in cheminformatics workflows, especially when the textual representations were not provided.

### Deployment of machine learning model as a Web-Based application

After model optimization, the best-performing model was deployed as a web application on the ENHPCG platform (https://enhpcg.sci.eg/bioactivity-services), (https://enhpcg.sci.eg/bioactivity-services) (https://enhpcgic50predection.streamlit.app/).

**Fig. 13:**
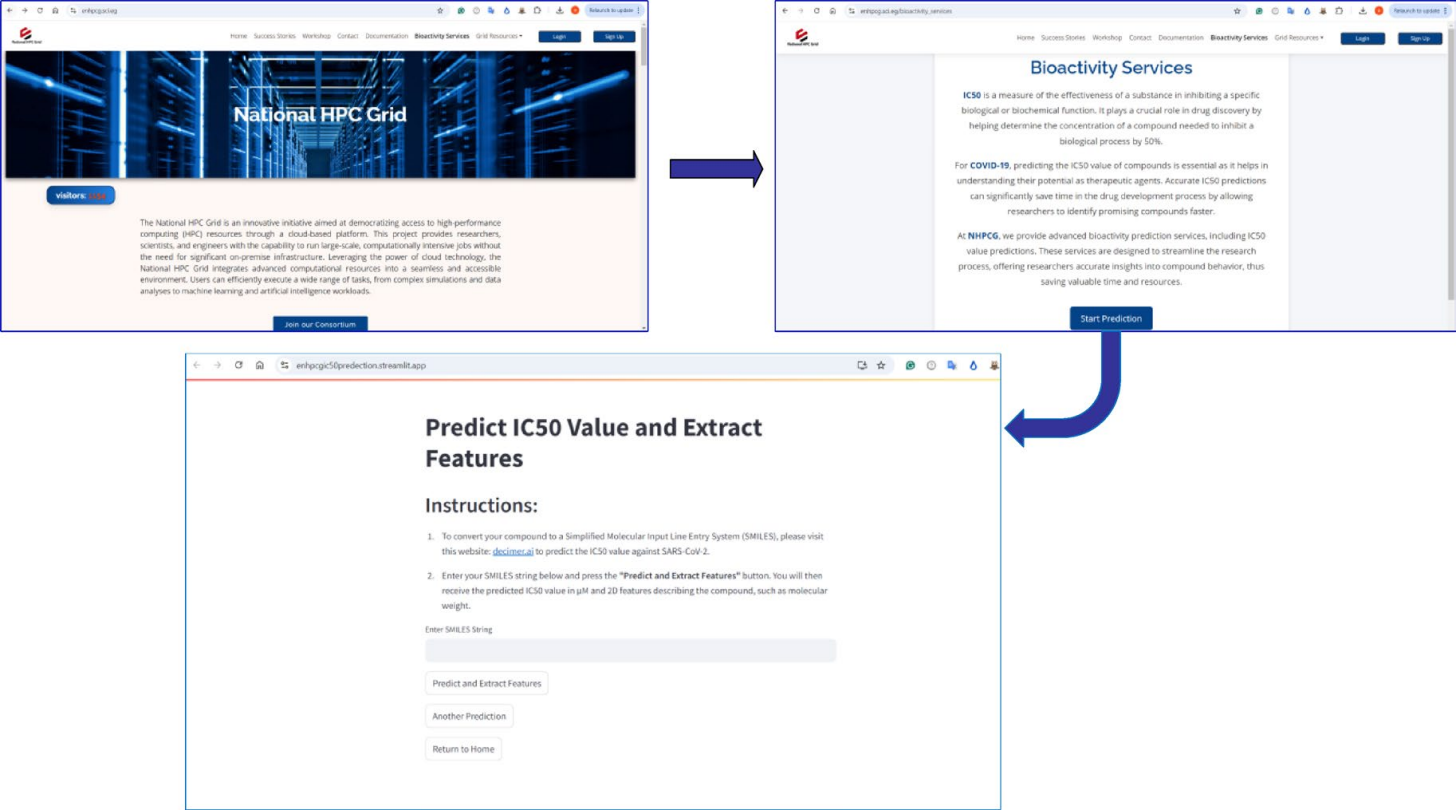
IC_50_ Prediction Application Hosted on the ENHPCG Platform.

As illustrated in Fig. 13, users can input molecules as SMILES, and the tool outputs predicted IC₅₀ values by transforming predicted pIC₅₀ via $\:IC_{50} = {10}^{-{pIC}_{50}}$. This user-friendly system enables rapid in-silico screening of antiviral compounds.

### Experimental validation

To validate predictive accuracy, we compared predicted IC₅₀ values of newly isolated echinoderm-derived compounds against their experimentally measured IC₅₀ values from in-vitro assays. This comparison confirmed the model’s reliability and potential applicability in real-world antiviral drug discovery pipelines.

### Statistical analysis

Statistical comparisons were conducted utilizing one-way analysis of variance (ANOVA) in SPSS version 15, with a significance level established at *p* < 0.05. Results are presented as mean ± standard deviation (SD).

## Supplementary Information

Below is the link to the electronic supplementary material.

Supplementary Material 1

## Data Availability

All data supporting the conclusions of this investigation are included within the main manuscript and Supplementary Information. Additional raw datasets are available from the corresponding author upon reasonable request. Source data are included.
