# Supplementary material for: Analytical and machine learning approaches identify a sea star steroid with promising activity for COVID-19 therapeutic development
Source: Sci Rep. 2025 Oct 7;15:35013. doi: 10.1038/s41598-025-20443-6 (PMC12504724; doi:10.1038/s41598-025-20443-6)
Supplement: Supplementary file 1 — Supplementary Material 1 [file 41598_2025_20443_MOESM1_ESM.docx]

**SUPPLEMENTAL FILE**

**Analytical and Machine Learning Approaches Identify a Sea Star Steroid with Promising Activity for COVID-19 Therapeutic Development**

Mohamed S. M. Abd El Hafez**^1,*^**, Aya I. Maiyza**^2^**, Hanan A. Hassan**^2^**, Sohila Osama**^3^**, Mohamed G. Seadawy**^4^**, Maha A. El Demellawy**^5,6^**, Doaa A. Ghareeb**^6,7,8^**

**^1^** National Institute of Oceanography and Fisheries, NIOF, Cairo, Egypt.

**^2^** Informatics Research Institute, City of Scientific Research and Technological Applications (SRTA-City), Egypt.

**^3^** Institute of Graduate Studies and Research, Alexandria University, Egypt.

**^4^** Biological Prevention Department, Egyptian Army, Egypt.

**^5^** Medical Biotechnology Department, Genetic Engineering and Biotechnology Research Institute, City of Scientific Research and Technological Applications (SRTA-City), Egypt.

**^6^** Center of Excellence for Drug Preclinical Studies (CE-DPS), Pharmaceutical and Fermentation Industry Development Center, City of Scientific Research and Technological Applications (SRTA-City), New Borg El Arab, Alexandria, Egypt.

**^7^** Bio-screening and preclinical trial lab, Biochemistry Department, Faculty of Science, Alexandria University, Alexandria, Egypt.

**^8^** Research Projects unit, Pharos University in Alexandria, Canal El Mahmoudia Street, Beside Green Plaza Complex 21648, Alexandria, Egypt.

**^*^**Corresponding Author: mohamedsaid80@yahoo.com

**ABSTRACT**

The pressing demand for safe and efficient COVID-19 treatments has intensified interest in natural products, especially those derived from marine organisms. In this study, a bioactive steroidal compound, 5α-cholesta-9(11)-en-3β,20β-diol, was successfully isolated from the starfish *Acanthaster planci*. Structural elucidation was achieved using HREIMS, FTIR, and advanced 1D/2D NMR spectroscopy, confirming a molecular formula of C₂₇H₄₆O₂ and characteristic functionalities including hydroxyl and double bond moieties. The compound demonstrated notable anti-SARS-CoV-2 activity, attaining 85% viral inhibition at 5 ng/μl with an IC₅₀ of 5.86 μM, as demonstrated by plaque reduction assays. Molecular docking studies demonstrated significant binding affinities toward key viral targets Mpro, NSP10, and RNA-dependent RNA polymerase with free binding energies of -26.85, -27.59, and -35.08 kcal/mol, respectively. These affinities surpassed those of their respective co-crystallized reference ligands. *In-silico* ADMET profiling indicated favorable pharmacokinetic properties, including high BBB penetration, moderate intestinal absorption, and non-hepatotoxicity. Toxicity assessments predicted low carcinogenic risk, a high rat MTD, and minimal ocular and dermal irritancy. Additionally, we developed a predictive web application based on machine learning to estimate IC₅₀ values of SARS-CoV-2 inhibitors, streamlining the drug discovery process. The forecasted values nearly matched the experimental outcomes, demonstrating the model’s reliability and its potential to reduce time, cost, and risk in early-stage drug development. Moreover, machine learning models, particularly XGBoost, demonstrated excellent performance in predicting pIC₅₀ values (RMSE = 0.1357, MAE = 0.1022), supporting the development of a web-based IC₅₀ prediction application. The bioactivity prediction platform ENHPCG further validated the compound’s antiviral potential, estimating an IC₅₀ of 5.95 μM. Overall, these integrated analytical, biological, and computational approaches highlight 5α-cholesta-9(11)-en-3β,20β-diol as a potential SARS-CoV-2 inhibitor and a candidate for further pharmacological development.

**Keywords:**

COVID-19 inhibitor

Marine natural products

Machine learning

Molecular docking

Drug discovery

**Supplemental Data**

**Fig. S1.** Flow chart of the isolated compound from starfish (*Acanthaster planci*).

**Fig. S2.** ^1^H NMR spectrum of the isolated compound (5α-cholesta-9(11)-en-3β, 20β-diol)

**Fig. S3.** ^13^C NMR spectrum of 5α-cholesta-9(11)-en-3β, 20β-diol

**Fig. S4.** HSQC spectrum of 5α-cholesta-9(11)-en-3β, 20β-diol

**Fig. S5.** HMBC spectrum of 5α-cholesta-9(11)-en-3β, 20β-diol

**Fig. S6.** COSY spectrum of 5α-cholesta-9(11)-en-3β, 20β-diol

**Fig. S7.** NOESY spectrum of 5α-cholesta-9(11)-en-3β, 20β-diol

**Fig. S8.** FT-IR spectrum of 5α-cholesta-9(11)-en-3β, 20β-diol

**Fig. S9.** (A) Three-dimensional of the isolated compound bound within the active site of the COVID-19 NSP10 protein. (B) Two-dimensional of the isolated compound docked into the NSP10 active site, overlaid with the co-crystallized ligand for comparison. (C) Surface mapping illustrating the spatial accommodation of the isolated compound within the active pocket of COVID-19 NSP10.
**Fig. S10.** Schematic diagram for the docking workflow.

**Table S1.** NMR spectroscopic data of the isolated compound from *Acanthaster planci* starfish.

**Table S2.** The binding free energies derived from molecular docking studies for the isolated compound and the corresponding co-crystallized compounds against selected SARS-CoV-2 target proteins.

**Table S3.** The predicted ADMET properties of the isolated compound in comparison with ritonavir.

**Table S4.** Comparative analysis between the experimental IC_50_ values reported by ^26^ and the values predicted by our machine learning model.

**Table S5.** Comparative analysis of the calculated IC_50_ values for selected compounds reported by ^27^ and the predicted IC_50_ values generated using the proposed ML model.

**Table S6.** Toxicity properties of the isolated compound.

Filtration

Concentration using Rotary evaporator (40°C)

Extraction by Chloroform / Methanol /one week / R.T

**starfish *(Acanthaster planci)***

**Crude extract** (**6.25** g)

**Fig. S1: Flow chart of the isolated compound from starfish (*Acanthaster planci*).**

White crystals

**Pure Compound (7.8** mg)

Chromatographed on silica gel (2^nd^ column)

Eluting with DCM-Methanol (95:5%)

Preparative TLC (DCM 95%: Acetone 5%)

**Fr.11**

**360 fractions then combined (fractions with the same RF value) to give**

**fractions (1 - 27)**

Chromatographed on silica gel (1^ry^ column)

Eluting with Hexane-DCM-Methanol


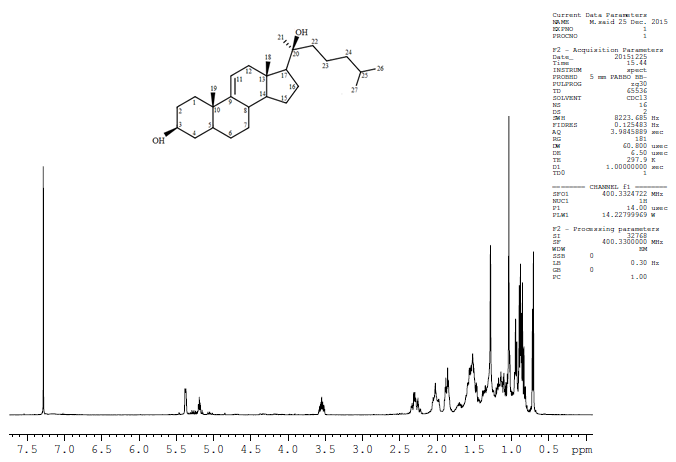


**Fig. S2. ^1^H NMR spectrum of 5α-cholesta-9(11)-en-3β, 20β-diol**


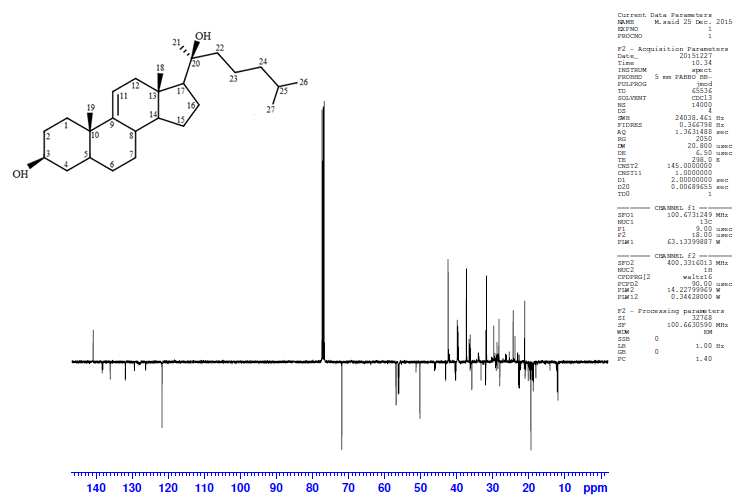


**Fig. S3. ^13^C NMR spectrum of 5α-cholesta-9(11)-en-3β, 20β-diol**


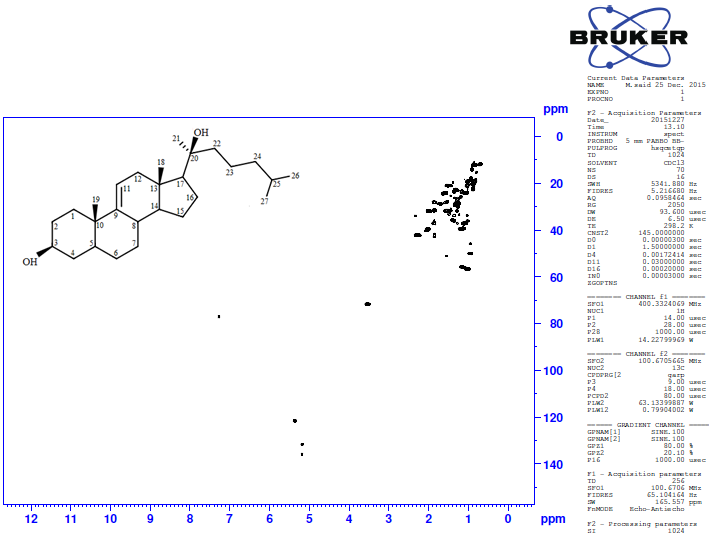


**Fig. S4. HSQC spectrum of 5α-cholesta-9(11)-en-3β, 20β-diol**


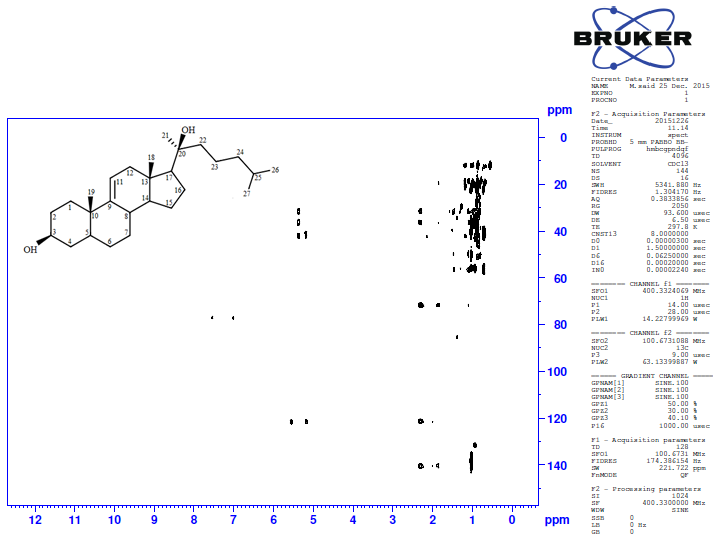


**Fig. S5. HMBC spectrum of 5α-cholesta-9(11)-en-3β, 20β-diol**


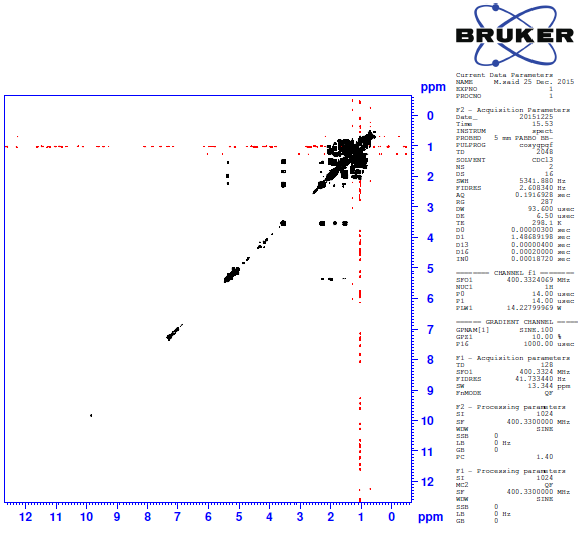


**Fig. S6. COSY spectrum of 5α-cholesta-9(11)-en-3β, 20β-diol**


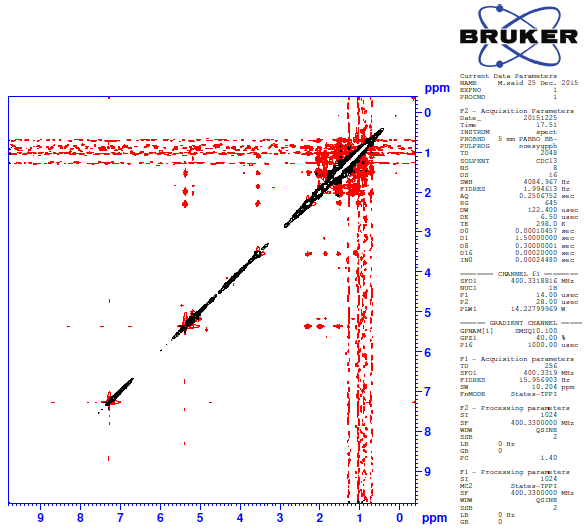


**Fig. S7. NOESY spectrum of 5α-cholesta-9(11)-en-3β, 20β-diol**

**
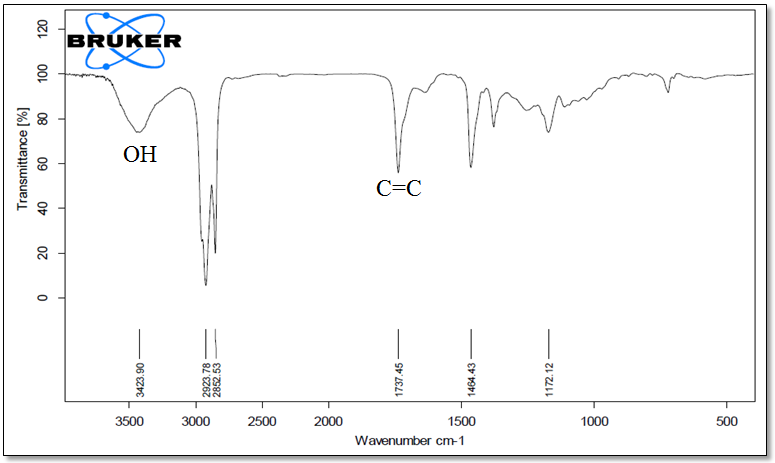
**

**Fig. S8. FT-IR spectrum of 5α-cholesta-9(11)-en-3β, 20β-diol**

| 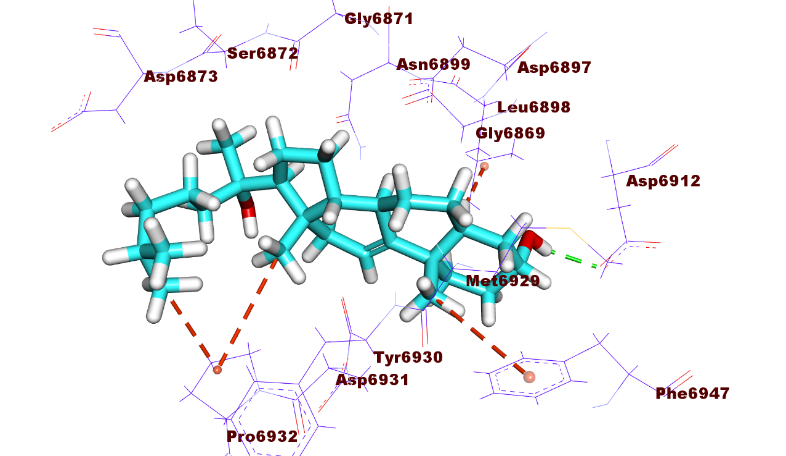 | | 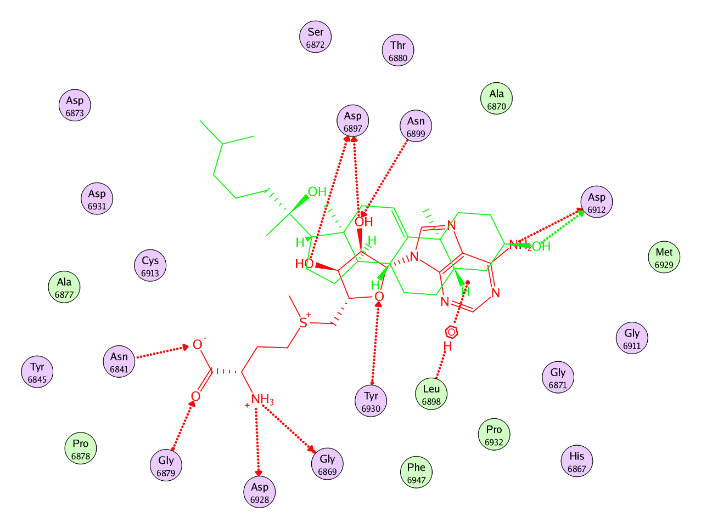 | |
| --- | --- | --- | --- |
| **(A)** | | **(B)** | |
| 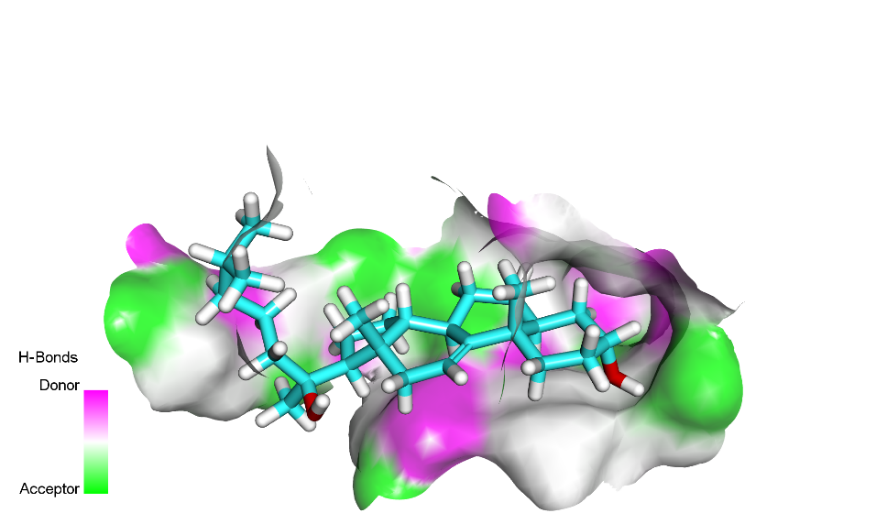 | |  |  |
| **(C)** | |  |  |

**Fig. S9.** (A) Three-dimensional of the isolated compound bound within the active site of the COVID-19 NSP10 protein. (B) Two-dimensional of the isolated compound docked into the NSP10 active site, overlaid with the co-crystallized ligand for comparison. (C) Surface mapping illustrating the spatial accommodation of the isolated compound within the active pocket of COVID-19 NSP10.


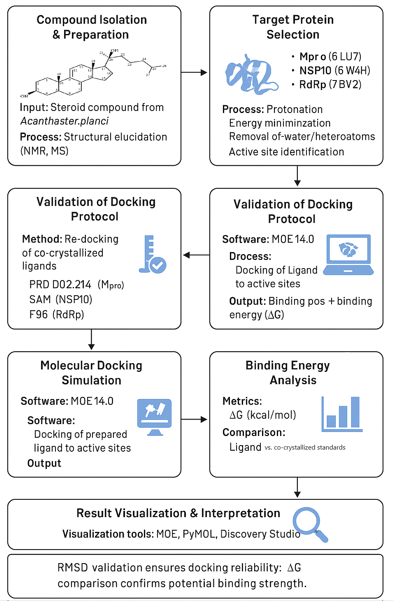


**Fig. S10. Schematic diagram for the docking workflow**

**Table S1.** NMR spectroscopic data of the isolated compound from *Acanthaster planci* starfish

| position | δ_H_ (*J* in HZ), mult. | δ_C_ |
| --- | --- | --- |
| 1 | 1.05(m) | 37.2 |
| 2 | 1.86(m) | 39.6 |
| 3 | 3.55(m) | 72.0 |
| 4 | 1.98(m) | 39.5 |
| 5 | 1.70(m) | 50.1 |
| 6 | 1.71(m) | 35.0 |
| 7 | 1.68(m) | 31.9 |
| 8 | 1.89(m) | 22.5 |
| 9 |  | 140.8 |
| 10 |  | 42.2 |
| 11 | 5.37(m) | 138.2 |
| 12 | 1.99(m) | 31.4 |
| 13 |  | 34.0 |
| 14 | 0.87(m) | 22.7 |
| 15 | 1.57(m) | 27.1 |
| 16 | 1.35(m) | 29.7 |
| 17 | 1.20(m) | 29.2 |
| 18 | 0.70(s) | 14.1 |
| 19 | 0.72(s) | 18.0 |
| 20 |  | 41.7 |
| 21 | 1.28(s) | 27.8 |
| 22 | 1.17(m) | 25.4 |
| 23 | 1.14(m) | 24.3 |
| 24 | 1.10(m) | 23.8 |
| 25 | 1.19(m) | 20.1 |
| 26 | 0.93(d,2) | 19.4 |
| 27 | 0.95(d,1.72) | 19.6 |

**^1^H NMR at 400 MHz, ^13^C NMR at 100 MHz in CDCl_3_**

**Table S2.** The binding free energies derived from molecular docking studies for the isolated compound and the corresponding co-crystallized compounds against selected SARS-CoV-2 target proteins.

| **Compound** | **COVID-19 main protease** | **NSP10** | **RNA-dependent RNA polymerase** |
| --- | --- | --- | --- |
|  | **ΔG (kcal/mol)** | | |
| The isolated Compound | **-26.85** | **-27.59** | **-35.08** |
| Co-crystallized ligand (PRD_002214) | -24.60 | - | - |
| Co-crystallized ligand (SAM) | - | -17.70 | - |
| Co-crystallized ligand (F86) | - | - | -23.56 |

**Table S3.** The predicted ADMET properties of the isolated compound in comparison with ritonavir.

| Comp. | BBB level ^a^ | Solubility level ^b^ | Absorption level ^c^ | CYP2D6 prediction ^d^ | Hepatotoxicity | PPB prediction ^e^ |
| --- | --- | --- | --- | --- | --- | --- |
| The isolated compound | 0 | 1 | 1 | false | false | true |
| Ritonavir | 4 | 2 | 2 | true | true | false |

^a^ BBB level, blood brain barrier level, 0 = very high, 1 = high, 2 = medium, 3 = low, 4 = very low.

^b^ Solubility level, 1 = very low, 2 = low, 3 = good, 4 = optimal.

^c^ Absorption level, 0 = good, 1 = moderate, 2 = poor, 3 = very poor.

^d^ CYP2D6, cytochrome P2D6, TRUE = inhibitor, FALSE = non inhibitor.

^e^ PBB, plasma protein binding, FALSE means less than 90%, TRUE means more than 90%

**Table S4.** Comparative analysis between the experimental IC50 values reported by ^26^ and the values predicted by our machine learning model.

| **ID** | **SMILES** | **IC_50_(μM)** | **Predicted IC_50_(μM)** |
| --- | --- | --- | --- |
| DS1 | CC1(C)[C@H]2C(C(=O)N[C@H](C=O)C[C@@H]3CCNC3=O)N(C[C@H]21)C(=O)c1cc2ccccc2cc1 | 0.453 | 0.145 |
| DS2 | CC1(C)[C@H]2C(C(=O)N[C@H](C=O)C[C@@H]3CCNC3=O)N(C[C@H]21)C(=O)COc1cc2ccccc2cc1 | 0.052 | 0.0482 |
| DS3 | CC1(C)[C@H]2C(C(=O)N[C@H](C=O)C[C@@H]3CCNC3=O)N(C[C@H]21)C(=O)CCc1cccc(F)c1 | 0.0165 | 0.0187 |
| DS4 | CC1(C)[C@@H]2C(C(=O)N[C@@H](C=O)C[C@@H]3CCNC3=O)N(C[C@@H]21)C(=O)COc1cc(F)cc(F)c1 | 0.185 | 0.0178 |
| DS5 | CC1(C)[C@H]2C(C(=O)N[C@H](C=O)C[C@@H]3CCNC3=O)N(C[C@H]21)C(=O)COc1ccc(F)c(F)c1 | 0.0132 | 0.0175 |
| DS6 | CC1(C)[C@@H]2C(C(=O)N[C@@H](C=O)C[C@H]3CCNC3=O)N(C[C@@H]21)C(=O)COc1ccc(OC)c(OC)c1 | 0.0145 | 0.0184 |
| DS7 | CC1(C)[C@H]2C(C(=O)N[C@H](C=O)C[C@@H]3CCNC3=O)N(C[C@H]21)C(=O)COc1cc(ccc1)C(F)(F)F | 0.0433 | 0.0225 |
| DS8 | CC1(C)[C@@H]2C(C(=O)N[C@@H](C=O)C[C@H]3CCNC3=O)N(C[C@@H]21)C(=O)COc1ccc(OC)c(F)c1 | 0.0372 | 0.0218 |
| DS9 | CC1(C)[C@H]2C(C(=O)N[C@H](C=O)C[C@@H]3CCNC3=O)N(C[C@H]21)C(=O)COc1ccc(OC(F)(F)F)cc1 | 0.0152 | 0.0225 |
| DS10 | CC1(C)[C@@H]2C(C(=O)N[C@@H](C=O)C[C@H]3CCNC3=O)N(C[C@@H]21)C(=O)COc1ccc(Cl)c(Br)c1 | 0.0508 | 0.0374 |
| DS11 | CC1(C)[C@@H]2C(C(=O)N[C@@H](C=O)C[C@@H]3CCNC3=O)N(C[C@@H]21)C(=O)COc1ccc(Br)cc1Cl | 0.0133 | 0.0374 |
| DS12 | CC1(C)[C@@H]2C(C(=O)N[C@@H](C=O)C[C@H]3CCNC3=O)N(C[C@@H]21)C(=O)COc1ccc(Cl)cc1 | 0.019 | 0.0174 |
| DS13 | CC1(C)[C@H]2C(C(=O)N[C@H](C=O)C[C@@H]3CCNC3=O)N(C[C@H]21)C(=O)COc1ccc(Cl)c(Cl)c1 | 0.0124 | 0.0172 |
| DS14 | CC1(C)[C@@H]2C(C(=O)N[C@@H](C=O)C[C@H]3CCNC3=O)N(C[C@@H]21)C(=O)COc1ccc(Cl)cc1Cl | 0.013 | 0.0172 |
| DS15 | O=C1NCC[C@@H]1C[C@H](C=O)NC(=O)C1[C@H]2CCC[C@H]2CN1C(=O)c1cccnc1 | 0.7485 | 0.5294 |
| DS16 | O=C1NCC[C@@H]1C[C@H](C=O)NC(=O)C1[C@H]2CCC[C@H]2CN1C(=O)c1cc2ccccc2[NH]1 | 0.1531 | 0.1432 |
| DS17 | O=C1NCC[C@@H]1C[C@H](C=O)NC(=O)C1[C@H]2CCC[C@H]2CN1C(=O)c1cc2ccccn2n1 | 0.2988 | 0.3812 |
| DS18 | O=C1NCC[C@@H]1C[C@H](C=O)NC(=O)C1[C@H]2CCC[C@H]2CN1C(=O)/C=C/c1ccccc1 | 0.5259 | 0.4086 |
| DS19 | O=C1NCC[C@@H]1C[C@H](C=O)NC(=O)C1[C@H]2CCC[C@H]2CN1C(=O)/C=C/c1ccc(OC)cc1OC | 0.1956 | 0.1988 |
| DS20 | CN(C)c1ccc(cc1)/C=C/C(=O)N1C[C@@H]2CCC[C@@H]2C1C(=O)N[C@@H](C=O)C[C@H]1CCNC1=O | 0.375 | 0.6716 |
| DS21 | O=C1NCC[C@@H]1C[C@H](C=O)NC(=O)C1[C@H]2CCC[C@H]2CN1C(=O)CCc1cccc(F)c1 | 0.0076 | 0.0116 |
| DS22 | O=C1NCC[C@@H]1C[C@H](C=O)NC(=O)C1[C@H]2CCC[C@H]2CN1C(=O)CCc1ccc(F)c(F)c1 | 0.0174 | 0.0173 |
| DS23 | O=C1NCC[C@@H]1C[C@H](C=O)NC(=O)C1[C@H]2CCC[C@H]2CN1C(=O)CCc1cc(F)cc(F)c1 | 0.0076 | 0.0116 |
| DS24 | CN(C)c1ccc(cc1)CCC(=O)N1C[C@@H]2CCC[C@@H]2C1C(=O)N[C@@H](C=O)C[C@H]1CCNC1=O | 0.3782 | 0.3481 |
| DS25 | O=C1NCC[C@@H]1C[C@H](C=O)NC(=O)C1[C@H]2CCC[C@H]2CN1C(=O)COc1ccc(OC)cc1 | 0.0362 | 0.0329 |
| DS26 | O=C1NCC[C@@H]1C[C@H](C=O)NC(=O)C1[C@H]2CCC[C@H]2CN1C(=O)C(C)Oc1ccc(OC)cc1 | 0.0691 | 0.0731 |
| DS27 | O=C1NCC[C@H]1C[C@@H](C=O)NC(=O)C1[C@@H]2CCC[C@@H]2CN1C(=O)COc1ccc2OCCOc2c1 | 0.0935 | 0.1092 |
| DS28 | O=C1NCC[C@H]1C[C@@H](C=O)NC(=O)C1[C@@H]2CCC[C@@H]2CN1C(=O)COc1ccc(Cl)cc1 | 0.0092 | 0.0143 |
| DS29 | O=C1NCC[C@H]1C[C@@H](C=O)NC(=O)C1[C@@H]2CCC[C@@H]2CN1C(=O)COc1ccc(Cl)cc1F | 0.0347 | 0.0201 |
| DS30 | O=C1NCC[C@H]1C[C@@H](C=O)NC(=O)C1[C@@H]2CCC[C@@H]2CN1C(=O)COc1ccc(Cl)cc1Cl | 0.0172 | 0.0279 |
| DS31 | O=C1NCC[C@H]1C[C@@H](C=O)NC(=O)C1[C@@H]2CCC[C@@H]2CN1C(=O)COc1ccc(Cl)c(Cl)c1 | 0.03 | 0.0279 |
| DS32 | O=C1NCC[C@H]1C[C@@H](C=O)NC(=O)C1[C@@H]2CCC[C@@H]2CN1C(=O)COc1ccc(Cl)cc1C | 0.0197 | 0.0221 |

**Table S5.** Comparative analysis of the calculated IC50 values for selected compounds reported by ^27^ and the predicted IC50 values generated using the proposed ML model.

| **Drug** | **Structure/SMILES** | **Calculated IC_50_** | **Predicted IC_50_** |
| --- | --- | --- | --- |
| Ebselen | 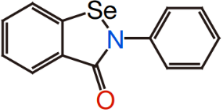  C1=CC=C(C=C1)N2C(=O)C3=CC=CC=C3[Se]2 | 0.67 ± 0.09 | 0.1198 |
| Disulfiram | 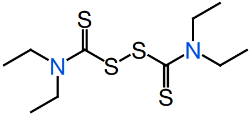  CCN(CC)C(=S)SSC(=S)N(CC)CC | 9.35 ± 0.18 | 2.9915 |
| Tideglusib | 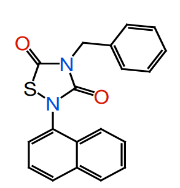  C1=CC=C(C=C1)CN2C(=O)N(C3=CC=CC4=CC=CC=C43)SC2=O | 1.55 ± 0.30 | 0.2774 |
| Carmofur | 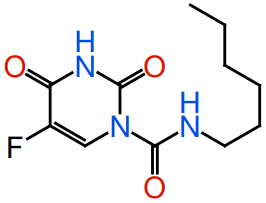  CCCCCCNC(=O)N1C=C(C(=O)NC1=O)F | 1.82 ± 0.06 | 2.2864 |
| Shikonin | 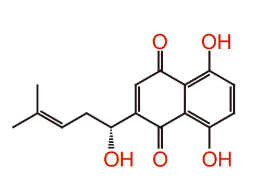  CC(=CC[C@H](C1=CC(=O)C2=C(C=CC(=C2C1=O)O)O)O)C | 15.75 ± 8.22 | 8.5240 |
| PX-12 | 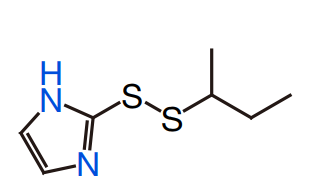  CCC(C)SSC1=NC=CN1 | 21.39 ± 7.06 | 6.6657 |

**Table S6.** Toxicity properties of the isolated compound.

| **Compound** | FDA Rodent Carcinogenicity  (Rat- Male) | Carcinogenic Potency TD_50_  (Rat) ^a^ | Rat Maximum Tolerated Dose  (Feed) ^b^ | Rat Oral LD_50_ ^b^ | Rat Chronic LOAEL ^b^ | Ocular Irritancy | Skin Irritancy |
| --- | --- | --- | --- | --- | --- | --- | --- |
| The isolated compound | Non-Carcinogen | 2.651 | 0.047 | 2.855 | 0.005 | Irritant | None |
| Ritonavir | Non-Carcinogen | 1.369 | 0.038 | 0.477 | 0.014 | Irritant | Moderate |

^a^ Unit: mg/kg body weight/day

^b^ Unit: g/kg body weight
